# Use of smoking cessation pharmacotherapies during pregnancy is not associated with increased risk of adverse pregnancy outcomes: a population-based cohort study

**DOI:** 10.1186/s12916-019-1472-9

**Published:** 2020-02-05

**Authors:** Duong Thuy Tran, David B. Preen, Kristjana Einarsdottir, Anna Kemp-Casey, Deborah Randall, Louisa R. Jorm, Stephanie K. Y. Choi, Alys Havard

**Affiliations:** 1 Centre for Big Data Research in Health, Faculty of Medicine, University of New South Wales (UNSW), Sydney, NSW 2052 Australia; 2 School of Population and Global Health, University of Western Australia, Perth, Australia; 3 Centre of Public Health Sciences, Faculty of Medicine, University of Iceland, Reykjavik, Iceland; 4 Quality Use of Medicines and Pharmacy Research Centre, University of South Australia, Adelaide, Australia; 5 The University of Sydney Northern Clinical School, Women and Babies Research, St Leonards, NSW Australia

**Keywords:** Adverse outcomes, Australia, Birth defects, Bupropion, Nicotine replacement therapy, Pregnancy, Smoking cessation pharmacotherapy, Smoking in pregnancy, Varenicline

## Abstract

**Background:**

Varenicline, bupropion and nicotine replacement therapy (NRT) are three effective pharmacotherapies for smoking cessation, but data about their safety in pregnancy are limited. We assessed the risk of adverse perinatal outcomes and major congenital anomalies associated with the use of these therapies in pregnancy in Australia.

**Methods:**

Perinatal data for 1,017,731 deliveries (2004 to 2012) in New South Wales and Western Australia were linked to pharmaceutical dispensing, hospital admission and death records. We identified 97,875 women who smoked during pregnancy; of those, 233, 330 and 1057 were exposed to bupropion, NRT and varenicline in pregnancy, respectively. Propensity scores were used to match exposed women to those who were unexposed to any smoking therapy (1:10 ratio). Propensity scores and gestational age at exposure were used to match varenicline-exposed to NRT-exposed women (1:1 ratio). Time-dependent Cox proportional hazards models estimated hazard ratios (HR) with 95% confidence intervals (95% CI) for any adverse perinatal event (a composite of 10 unfavourable maternal and neonatal outcomes) and any major congenital anomaly.

**Results:**

The risk of any adverse perinatal event was not significantly different between bupropion-exposed and unexposed women (39.2% versus 39.3%, HR 0.93, 95% CI 0.73–1.19) and between NRT-exposed and unexposed women (44.8% vs 46.3%, HR 1.02, 95% CI 0.84–1.23), but it was significantly lower in women exposed to varenicline (36.9% vs 40.1%, HR 0.86, 95% CI 0.77–0.97). Varenicline-exposed infants were less likely than unexposed infants to be born premature (6.5% vs 8.9%, HR 0.72, 95% CI 0.56–0.92), be small for gestational age (11.4% vs 15.4%, HR 0.68, 95% CI 0.56–0.83) and have severe neonatal complications (6.6% vs 8.2%, HR 0.74, 95% CI 0.57–0.96). Among infants exposed to varenicline in the first trimester, 2.9% had a major congenital anomaly (3.5% in unexposed infants, HR 0.91, 95% CI 0.72–1.15). Varenicline-exposed women were less likely than NRT-exposed women to have an adverse perinatal event (38.7% vs 51.4%, HR 0.58, 95% CI 0.33–1.05).

**Conclusions:**

Pregnancy exposure to smoking cessation pharmacotherapies does not appear to be associated with an increased risk of adverse birth outcomes. Lower risk of adverse birth outcomes in varenicline-exposed pregnancies is inconsistent with recommendations that NRT be used in preference to varenicline.

## Introduction

Smoking cessation in pregnancy reduces the risk of perinatal adverse outcomes and has long-term maternal and child health benefits [1, 2]. However, a substantial proportion of women continue to smoke throughout gestation [3] and even in subsequent pregnancies despite having prior poor birth outcomes [4]. This illustrates the difficulty of quitting for women who have high levels of nicotine dependence [5], and highlights the need for effective assistance. Pharmacotherapies for smoking cessation including varenicline, bupropion and nicotine replacement therapy (NRT) are more effective than behavioural cessation interventions in non-pregnant adults [6, 7]; however, little is known about their safety and efficacy in pregnancy [1, 8–10].

Research evidence about pregnancy outcomes associated with varenicline exposure is limited to uncontrolled and small studies, including a case series for 24 women [11, 12] and 89 other cases identified from teratology counselling and surveillance services [13]. Available data regarding pregnancy outcomes of bupropion for smoking cessation (i.e. not for depression) are limited to two small pilot trials (*n* = 35) [14, 15] and two small observational studies (*n* < 140) [16, 17]. Given the lack of robust evidence regarding their safety, varenicline and bupropion have no therapeutic indications for smoking cessation in pregnancy [2, 9, 10]. For NRT on the other hand, there have been trials [1, 18] and large-scale observational studies [1, 19–21]; however, neither have been conclusive [1, 18–21]. This is most likely due to poor adherence to NRT [1] and heterogeneity in the NRT products (e.g. patches, gums, lozenges, spray) under investigation [19–21]. Transdermal NRT releases continuous doses of nicotine, thus is potentially associated with greater risk of harm than intermittent doses offered by oral forms. Clinical guidelines support the use of NRT during pregnancy only when the expected benefits outweigh the potential risks [2, 9, 10].

Although NRT clinical trials have been possible, based on the assumption that NRT is safer than continued smoking in pregnancy, this has not been the case for varenicline or bupropion. In the absence of sufficient evidence, we established the Smoking MUMS (Maternal Use of Medications and Safety) Study [22], a population-based cohort study focused on smoking cessation pharmacotherapies during pregnancy. In this Australian cohort of women who smoked during pregnancy, 3.6% used a smoking cessation therapy, mostly varenicline (1.8%) and NRT (1.7%) [23]. The current paper aimed to:

1. Compare the risk of adverse perinatal outcomes and major congenital anomalies between pregnancies exposed to these pharmacotherapies and pregnancies exposed to smoking but no pharmacotherapy and
2. Compare the risk of these outcomes between pregnancies that were exposed to different therapies.

## Methods

### Study data source

The Smoking MUMS Study is a cohort design based on all pregnancies that resulted in a birth (gestational age ≥ 20 weeks or birthweight ≥ 400 g) in two Australian states, New South Wales (NSW) and Western Australia (WA), between 2003 and 2012. The study protocol, data sources and data preparation have been described elsewhere [22, 24]. The current analyses used linked records from four data sources including perinatal data (deliveries in 2003–2012), dispensing data for pharmaceuticals subsidised through the Pharmaceutical Benefits Scheme (PBS, 2003–2013), hospital admissions (2001–2013) and deaths (2003–2014). Under Australian universal health care system, eligible residents had access to subsidised prescriptions of bupropion (for smoking cessation only) from February 2001 and varenicline from January 2008. Subsidy for NRT patches commenced in December 2008, initially only for Aboriginal and Torres Strait Islander people, and then for the general population since January 2011. At time of conducting this study, oral forms of NRT were not subsidised in Australia. All forms of NRT, including patches, were also available over the counter whilst bupropion and varenicline were only available on prescription.

### Study population

We identified a base cohort of pregnant women (conception between 1 January 2004 and 9 April 2012) who smoked during pregnancy. Conception date was estimated using the equation: date of conception = date of delivery − gestational age at delivery × 7 + 14 days [22]. The beginning and end points of cohort entry ensure that dispensing data were available for at least 1 year prior to conception and deliveries in 2012 did not disproportionately include pregnancies with gestation shorter than 40 weeks.

Maternal smoking status in pregnancy was derived from either perinatal or maternal hospital admission data [25]. During the study period, there were changes in maternal smoking items in the perinatal data collections (2010 in WA and 2011 in NSW). Therefore, for those who delivered before 2010 in WA and before 2011 in NSW, a woman was identified as having smoked during pregnancy if the response was Yes to the item about whether a woman smoked during pregnancy. For those who delivered in 2010 onwards in WA, smoking status was defined as Yes if the reported quantity of tobacco cigarettes smoked each day during the first or the second half of pregnancy was greater than 0. For those who delivered in NSW in 2011 onwards, smoking status was based on a Yes response to items indicating whether a woman smoked in the first or the second half of the pregnancy. Based on the hospital admission corresponding to the delivery [25], a woman was identified as having smoked during pregnancy if a Z72.0 code (i.e. use of tobacco in the last month) [26] was recorded in any diagnosis field. In addition, some women who were not identified as having smoked in pregnancy but had dispensing of a smoking cessation therapy during pregnancy were reclassified as smokers according to a published algorithm [23].

**Fig. 1:**
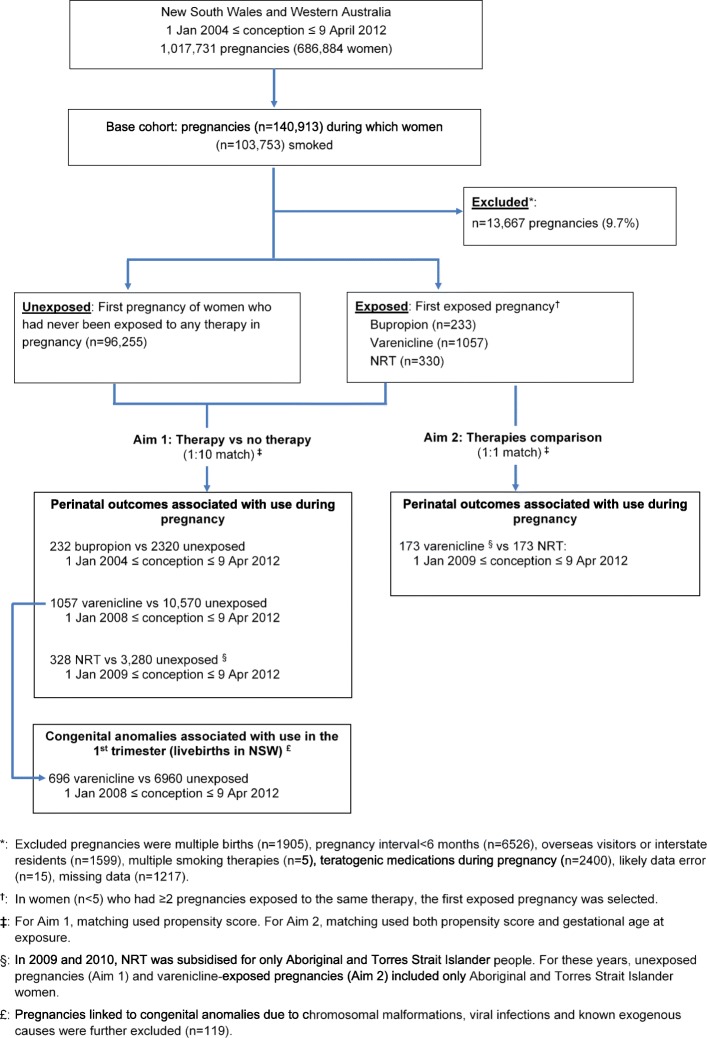
Flowchart of pregnancies included in the analyses

From the base cohort above, we identified women who were exposed to bupropion, varenicline and NRT during pregnancy and women who had never been exposed to any of these medicines in pregnancy. As aforementioned, subsidy for these medicines commenced at different time points. Accordingly, for comparisons involving bupropion, varenicline and NRT, the study periods started from 1 January 2004, 1 January 2008 and 1 January 2009, respectively. When comparing outcomes of different therapies (aim 2), we required exposure to occur over the same calendar time to avoid confounding by the underlying temporal variation in pregnancy and labour care [27, 28]. As only 30 pregnancies were exposed to bupropion between 2009 and 2012 (when both varenicline and NRT were available), comparisons between bupropion and other therapies were not conducted. In accordance with ethical approvals for this study, congenital anomalies were examined among infants born in NSW only. Cell sizes were such that this outcome could be examined among only varenicline-exposed pregnancies relative to pregnancies exposed to smoking but no pharmacotherapy (Fig. 1).

### Exposure to smoking cessation pharmacotherapy

Dispensing data included records of all subsidised varenicline (Anatomical Therapeutic Chemical [ATC] code N07BA03), NRT patches (N07BA01) and bupropion (code change from N07BA02 to N06AX12) [29]. The recommended course for non-pregnant adults is at least 7 weeks for bupropion (150 mg once daily for the first 3 days then 150 mg twice daily), at least 8 weeks for NRT (one patch daily) and 12 weeks for varenicline (0.5 mg once daily for the first 3 days then 1 mg once daily); one course generally involves two or more prescriptions [2]. Records of these therapies dispensed in the period from 100 days pre-conception to date of delivery were identified; the earliest dispensing in this period was referred to as the index dispensing. Days covered by each dispensing was estimated by dividing the quantity dispensed by recommended daily dosage. A pregnancy was identified as exposed if there were one or more dispensings of the therapy in the gestation period (i.e. conception to delivery) or pre-conception dispensings were sufficient to last into the gestation period (i.e. date of the index dispensing + days covered by pre-conception dispensings ≥ date of conception). Date of exposure was defined as either (i) date of the index dispensing if dispensed after conception or (ii) date of conception if pre-conception dispensings were sufficient to last into the gestation period or (iii) date of the first post-conception dispensing if pre-conception dispensings were insufficient to last into the gestation period. Gestational age at exposure (expressed as weeks, the whole number) was calculated using the formula [gestational age at delivery − (date of delivery − date of exposure)/7].

### Study outcomes

The two primary outcomes of the study were whether a woman or neonate experienced any adverse perinatal event at birth and whether the neonate had any major congenital anomaly. Any adverse perinatal event was a composite of 10 individual birth outcomes, including preterm birth (< 37 weeks, medically indicated or spontaneous), small for gestational age (SGA, birthweight < 10th percentile sex- and gestational age-specific) [30], Apgar score at 5 min < 7, admission to neonatal special care (NSC), severe neonatal morbidity complications [31], emergency caesarean section, severe maternal morbidity complications [32], preterm premature rupture of membranes (PPROM), placental abruption and perinatal death (stillbirth or 28-day neonatal death). These outcome variables were derived from the perinatal record, hospital record relating to the mother’s delivery, hospital record relating to the baby’s birth and mortality data. Outcomes including SGA, NSC admission, Apgar score and severe neonatal morbidity complications were assessed among live births only. The second primary outcome was a diagnosis of any major congenital anomaly recorded in hospital admissions occurring within 18 months from birth, among live-born babies in NSW. A detailed description of the study outcome variables is presented in Additional file 1.

### Exclusions

Exclusion criteria included multiple births, conception within 6 months from the immediate preceding delivery [33, 34], interstate residents and overseas visitors (likely incomplete capture of hospital admission and dispensing data), use of multiple smoking therapies in the same pregnancy, use of potentially teratogenic medications during pregnancy (category D and X, according to the Australian Therapeutic Goods Administration classification system) [35], likely data errors (birthweight < 1000 g whilst gestational age > 38 weeks, birthweight > 5500 g whilst gestational age < 37 week, based on the Australian birthweight chart) [30] and missing data (mostly due to geographical area of residence and Apgar score not being recorded). In the analysis of the major congenital anomaly outcome, pregnancies linked to anomalies due to chromosomal malformations, viral infection (e.g. cytomegalovirus, rubella) and known exogenous causes were also excluded. The numbers of excluded pregnancies according to exclusion criteria are presented in Fig. 1 and Additional file 1: Table S1.4.

### Statistical analyses

For aim 1 comparisons, among unexposed women, 24.5% had two or more pregnancies; their first pregnancy was selected. In the exposed groups, a small number of women (*n* < 5) had two pregnancies exposed to the same therapy; their first exposed pregnancy was selected. In the analyses for aim 2, there was no woman with a pregnancy exposed to both varenicline and NRT.

Propensity scores were calculated using logistic regression models in which the outcome variable was exposure to a smoking cessation therapy and explanatory variables included state of birth, year of conception, maternal age at conception, Aboriginal and/or Torres Strait Islander status, country of birth, marital status, quintiles of socio-economic disadvantage scores associated with the residential area [36], geographical remoteness of the residential area [37], private health insurance, parity, previous caesarean section, number of hospital admissions in the year prior to conception and pre-existing maternal morbidities (mental health, chronic airway conditions, diabetes, hypertension, cardiovascular diseases, epilepsy, chronic renal diseases, thyroid disorders, substance and alcohol diagnoses, anaemia and coagulation disorders, the use of steroids, non-steroid anti-inflammatory drugs and medications for gastro-oesophageal reflux diseases). For the analysis of congenital anomalies, the propensity score model included an additional variable indicating whether the mother had previously had a child with a major congenital anomaly. See Additional file 1 for a comprehensive description of variables included in propensity score models and how they were derived from the source data.

For analyses addressing aim 1, each exposed pregnancy was matched to ten unexposed pregnancies on propensity score, using a greedy five- to one-digit algorithm (matching on the five digits of propensity score first, then four digits if there was no five-digit match, and so on to one-digit matching, with no replacement) [38]. To address aim 2, the matching (1:1 ratio) used both propensity score and gestational age at exposure. Absolute standardised differences were calculated to assess balance in the characteristics of the comparison groups (balanced if the difference < 0.1) [39]. Cox proportional hazard modelling (discrete tier for matched data, gestational week as time scale) was used to estimate hazard ratios (HRs). In these Cox models, exposure was defined as a time-dependent variable, i.e. a pregnancy was considered unexposed until the gestational week at exposure. The window of exposure was the first trimester (gestational week at exposure < 13) when examining the congenital anomaly outcome, up to week 37 (gestational week at exposure < 37) for the composite adverse perinatal event and preterm birth, and until delivery for other individual perinatal outcomes.

For comparisons addressing aim 1, the matched samples were well-balanced on maternal characteristics (Table 1); thus, univariable Cox models were built. For aim 2 comparisons, multivariable Cox models were built to adjust for characteristics with a standardised difference > 0.1 [39]; however, only the multivariable model assessing the composite perinatal outcome converged. Therefore, for this composite perinatal outcome, both univariable and multivariable HRs were reported, and for individual perinatal outcomes, univariable HRs were presented.

**Table 1:** Demographic and clinical characteristics of exposed women and matched comparison group, stratified by therapy comparison. Data are numbers (percentages) and standardised differences

	Aim 1												Aim 2		
	Bupropion vs unexposed			Varenicline vs unexposed			NRT vs unexposed			Varenicline 1st trimester vs unexposed			Varenicline vs NRT		
	Bupropion	Unexposed	Diff*	Varenicline	Unexposed	Diff*	NRT	Unexposed^†^	Diff*	Varenicline	Unexposed	Diff*	Varenicline^†^	NRT	Diff*
Total number	232	2320		1057	10,570		328	3280		696	6960		173	173	
State of delivery															
New South Wales	177 (76.3%)	1819 (78.4%)	0.05	801 (75.8%)	8109 (76.7%)	0.02	248 (75.6%)	2484 (75.7%)	0.00	696 (100%)	6960 (100%)		131 (75.7%)	127 (73.4%)	0.05
Western Australia	55 (23.7%)	501 (21.6%)	0.05	256 (24.2%)	2461 (23.3%)	0.02	80 (24.4%)	796 (24.3%)	0.00				42 (24.3%)	46 (26.6%)	0.05
Year of conception															
2004	39 (16.8%)	389 (16.8%)	0.00												
2005	59 (25.4%)	613 (26.4%)	0.02												
2006	42 (18.1%)	391 (16.9%)	0.03												
2007	45 (19.4%)	483 (20.8%)	0.04												
2008	17 (7.3%)	194 (8.4%)	0.04	179 (16.9%)	1699 (16.1%)	0.02				116 (16.7%)	1057 (15.2%)	0.04			
2009	7 (3.0%)	75 (3.2%)	0.01	270 (25.5%)	2666 (25.2%)	0.01	9 (2.7%)	73 (2.2%)^†^	0.03	169 (24.3%)	1740 (25.0%)	0.02			
2010	12 (5.2%)	93 (4.0%)	0.06	315 (29.8%)	3153 (29.8%)	0.00	47 (14.3%)	412 (12.6%)^†^	0.05	220 (31.6%)	2200 (31.6%)	0.00	21 (12.2%)^†, §^	22 (12.7%) ^§^	0.02
2011	11 (4.7%)^#^	82 (3.6%)^#^	0.05	239 (22.6%)	2480 (23.5%)	0.02	212 (64.6%)	2228 (67.9%)	0.07	155 (22.3%)	1643 (23.6%)	0.03	123 (71.1%)	116 (67.1%)	0.09
2012				54 (5.1%)	572 (5.4%)	0.01	60 (18.3%)	567 (17.3%)	0.03	36 (5.2%)	320 (4.6%)	0.03	29 (16.8%)	35 (20.2%)	0.09
Maternal age (conception)															
Under 25	61 (26.3%)	589 (25.4%)	0.02	332 (31.4%)	3341 (31.6%)	0.00	94 (28.7%)	910 (27.7%)	0.02	215 (30.9%)	2172 (31.2%)	0.01	57 (32.9%)	47 (27.2%)	0.13
25–29	76 (32.8%)	792 (34.1%)	0.03	318 (30.1%)	3132 (29.6%)	0.01	99 (30.2%)	1060 (32.3%)	0.05	230 (33.0%)	2177 (31.3%)	0.04	53 (30.6%)	54 (31.2%)	0.01
30–34	61 (26.3%)	602 (25.9%)	0.01	227 (21.5%)	2360 (22.3%)	0.02	84 (25.6%)	819 (25.0%)	0.01	133 (19.1%)	1418 (20.4%)	0.03	34 (19.7%)	41 (23.7%)	0.10
35 and older	34 (14.7%)	337 (14.5%)	0.00	180 (17.0%)	1737 (16.4%)	0.02	51 (15.5%)	491 (15.0%)	0.02	118 (17.0%)	1193 (17.1%)	0.00	29 (16.8%)	31 (17.9%)	0.03
Aboriginal	9 (3.9%)	110 (4.7%)	0.04	101 (9.6%)	926 (8.8%)	0.03	93 (28.4%)	875 (26.7%)	0.04	71 (10.2%)	648 (9.3%)	0.03	32 (18.5%)	36 (20.8%)	0.06
Overseas born	35 (15.1%)	367 (15.8%)	0.02	180 (17.0%)	1815 (17.2%)	0.00	24 (7.3%)	206 (6.3%)	0.04	104 (14.9%)	1015 (14.6%)	0.01	26 (15.0%)	11 (6.4%)	0.28
Had a partner	173 (74.6%)	1764 (76.0%)	0.03	719 (68.0%)	7218 (68.3%)	0.01	179 (54.6%)	1792 (54.6%)	0.00	452 (64.9%)	4584 (65.9%)	0.02	119 (68.8%)	91 (52.6%)	0.34
Private health insurance	34 (14.7%)	288 (12.4%)	0.07	141 (13.3%)	1435 (13.6%)	0.01	23 (7.0%)	207 (6.3%)	0.03	87 (12.5%)	836 (12.0%)	0.01	20 (11.6%)	10 (5.8%)	0.21
Socio-economic disadvantage^‡^															
Quintile 1 (most disadvantaged)	48 (20.7%)	519 (22.4%)	0.04	172 (16.3%)	1722 (16.3%)	0.00	53 (16.2%)	479 (14.6%)	0.04	123 (17.7%)	1264 (18.2%)	0.01	33 (19.1%)	26 (15.0%)	0.11
Quintile 2	39 (16.8%)	411 (17.7%)	0.02	164 (15.5%)	1629 (15.4%)	0.00	62 (18.9%)	644 (19.6%)	0.02	112 (16.1%)	1073 (15.4%)	0.02	23 (13.3%)	41 (23.7%)	0.27
Quintile 3	46 (19.8%)	445 (19.2%)	0.02	263 (24.9%)	2546 (24.1%)	0.02	84 (25.6%)	875 (26.7%)	0.02	201 (28.9%)	1948 (28.0%)	0.02	40 (23.1%)	37 (21.4%)	0.04
Quintile 4	54 (23.3%)	524 (22.6%)	0.02	289 (27.3%)	3024 (28.6%)	0.03	91 (27.7%)	873 (26.6%)	0.03	173 (24.9%)	1763 (25.3%)	0.01	44 (25.4%)	46 (26.6%)	0.03
Quintile 5 (least disadvantaged)	45 (19.4%)	421 (18.1%)	0.03	169 (16.0%)	1649 (15.6%)	0.01	38 (11.6%)	409 (12.5%)	0.03	87 (12.5%)	912 (13.1%)	0.02	33 (19.1%)	23 (13.3%)	0.16
Remoteness of residence															
Major cities	135 (58.2%)	1374 (59.2%)	0.02	592 (56.0%)	5927 (56.1%)	0.00	170 (51.8%)	1677 (51.1%)	0.01	440 (63.2%)	4451 (64.0%)	0.02	96 (55.5%)	93 (53.8%)	0.03
Inner regional	63 (27.2%)	598 (25.8%)	0.03	339 (32.1%)	3474 (32.9%)	0.02	118 (36.0%)	1257 (38.3%)	0.05	200 (28.7%)	1999 (28.7%)	0.00	57 (32.9%)	62 (35.8%)	0.06
Outer regional, remote, very remote	34 (14.7%)	348 (15.0%)	0.01	126 (11.9%)	1169 (11.1%)	0.03	40 (12.2%)	346 (10.5%)	0.05	56 (8.0%)	510 (7.3%)	0.03	20 (11.6%)	18 (10.4%)	0.04
Parity															
Nulliparous	65 (28.0%)	699 (30.1%)	0.05	299 (28.3%)	3022 (28.6%)	0.01	73 (22.3%)	751 (22.9%)	0.02	221 (31.8%)	2197 (31.6%)	0.00	46 (26.6%)	41 (23.7%)	0.07
Multiparous (1 to 4)	152 (65.5%)	1466 (63.2%)	0.05	709 (67.1%)	7122 (67.4%)	0.01	237 (72.3%)	2367 (72.2%)	0.00	455 (65.4%)	4587 (65.9%)	0.01	116 (67.1%)	122 (70.5%)	0.07
Grand multiparous (≥ 5)	15 (6.5%)	155 (6.7%)	0.01	49 (4.6%)	426 (4.0%)	0.03	18 (5.5%)	162 (4.9%)	0.02	20 (2.9%)	176 (2.5%)	0.02	11 (6.4%)	10 (5.8%)	0.02
Previous caesarean section	33 (14.2%)	290 (12.5%)	0.05	175 (16.6%)	1663 (15.7%)	0.02	69 (21.0%)	626 (19.1%)	0.05	109 (15.7%)	1048 (15.1%)	0.02	28 (16.2%)	37 (21.4%)	0.13
Hospitalisation in 12 months prior															
Nil	187 (80.6%)	1901 (81.9%)	0.03	856 (81.0%)	8655 (81.9%)	0.02	254 (77.4%)	2608 (79.5%)	0.05	560 (80.5%)	5754 (82.7%)	0.06	142 (82.1%)	132 (76.3%)	0.14
Once	35 (15.1%)	327 (14.1%)	0.03	151 (14.3%)	1418 (13.4%)	0.03	54 (16.5%)	502 (15.3%)	0.03	101 (14.5%)	870 (12.5%)	0.06	23 (13.3%)	27 (15.6%)	0.07
Two or more	10 (4.3%)	92 (4.0%)	0.02	50 (4.7%)	497 (4.7%)	0.00	20 (6.1%)	170 (5.2%)	0.04	35 (5.0%)	336 (4.8%)	0.01	8 (4.6%)	14 (8.1%)	0.14
Morbidities															
Mental health	57 (24.6%)	534 (23.0%)	0.04	229 (21.7%)	2162 (20.5%)	0.03	131 (39.9%)	1224 (37.3%)	0.05	156 (22.4%)	1502 (21.6%)	0.02	39 (22.5%)	75 (43.4%)	0.45
Chronic airway	45 (19.4%)	416 (17.9%)	0.04	168 (15.9%)	1419 (13.4%)	0.07	57 (17.4%)	516 (15.7%)	0.04	119 (17.1%)	1069 (15.4%)	0.05	22 (12.7%)	28 (16.2%)	0.10
Gastro-oesophageal reflux	16 (6.9%)	149 (6.4%)	0.02	66 (6.2%)	585 (5.5%)	0.03	13 (4.0%)	137 (4.2%)	0.01	56 (8.0%)	486 (7.0%)	0.04	11 (6.4%)	8 (4.6%)	0.08
Use of NSAIDS	23 (9.9%)	206 (8.9%)	0.04	61 (5.8%)	510 (4.8%)	0.04	23 (7.0%)	197 (6.0%)	0.04	54 (7.8%)	481 (6.9%)	0.03	7 (4.0%)	9 (5.2%)	0.06
Use of steroids	11 (4.7%)	92 (4.0%)	0.04	30 (2.8%)	243 (2.3%)	0.03	14 (4.3%)	104 (3.2%)	0.06	25 (3.6%)	239 (3.4%)	0.01	< 5	5 (2.9%)	0.04
Anaemia and coagulation	10 (4.3%)	108 (4.7%)	0.02	26 (2.5%)	223 (2.1%)	0.02	13 (4.0%)	124 (3.8%)	0.01	16 (2.3%)	141 (2.0%)	0.02	5 (2.9%)	9 (5.2%)	0.12
Drug and alcohol disorder	7 (3.0%)	59 (2.5%)	0.03	25 (2.4%)	230 (2.2%)	0.01	30 (9.1%)	251 (7.7%)	0.05	19 (2.7%)	169 (2.4%)	0.02	5 (2.9%)	21 (12.1%)	0.36
Thyroid	< 5	21 (0.9%)	0.04	8 (0.8%)	79 (0.7%)	0.00	5 (1.5%)	52 (1.6%)	0.00	8 (1.1%)	62 (0.9%)	0.03	< 5	< 5	
Cardiovascular	< 5	5 (0.2%)	0.04	12 (1.1%)	113 (1.1%)	0.01	6 (1.8%)	70 (2.1%)	0.02	10 (1.4%)	87 (1.3%)	0.02	< 5	5 (2.9%)	0.18
Pre-existing diabetes	< 5	12 (0.5%)	0.01	13 (1.2%)	114 (1.1%)	0.01	5 (1.5%)	45 (1.4%)	0.01	< 5	55 (0.8%)	0.03	< 5	5 (2.9%)	0.04
Pre-existing hypertension	< 5	12 (0.5%)	0.04	11 (1.0%)	96 (0.9%)	0.01	6 (1.8%)	45 (1.4%)	0.04	6 (0.9%)	57 (0.8%)	0.00	< 5	< 5	0.15
Epilepsy	< 5	9 (0.4%)	0.01	8 (0.8%)	64 (0.6%)	0.02	8 (2.4%)	65 (2.0%)	0.03	5 (0.7%)	60 (0.9%)	0.02	< 5	5 (2.9%)	0.18
Chronic renal disease	< 5	27 (1.2%)	0.03	5 (0.5%)	42 (0.4%)	0.01	< 5	43 (1.3%)	0.01	< 5	30 (0.4%)	0.02	< 5	< 5	0.06

*Absolute standardised difference

^#^Data for 2011 and 2012 were combined due to frequency of women exposed to bupropion in 2012 was < 5

^†^For 2009 and 2010, only Aboriginal and Torres Strait Islander women were included

^§^Data for 2009 and 2010 were combined due to frequency of women exposed to NRT in 2009 was < 5

^‡^Quintile scores of the Index of Relative Socio-economic Disadvantage, based on residential area of women

< 5 In accordance with ethical approvals, frequencies less than 5 were not reported

Given the concern that women might have ceased therapy when they became aware of their pregnancy, we conducted sensitivity analyses by restricting the analyses to women who initiated the therapy after week 4 of gestation. It was only possible to conduct sensitivity analyses for the composite adverse perinatal event outcome (see Additional file 2: Table S2.6) because of small sample sizes for the other individual perinatal outcomes. All analyses were carried out in SAS version 9.4.

## Results

**Fig. 2:**
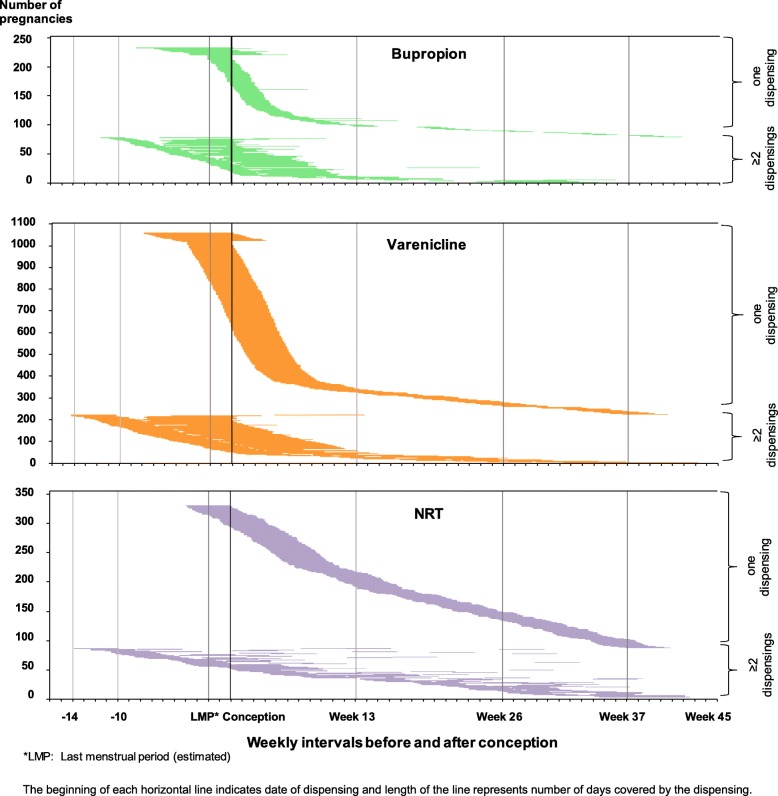
Timing and duration of exposure to a smoking cessation pharmacotherapy in pregnancy. The beginning of each horizontal line indicates the date of dispensing and the length of the line represents the number of days covered by the dispensing. *LMP last menstrual period (estimated)

In the Smoking MUMS Study, there were 1,017,731 pregnancies belonging to 686,884 women in NSW and WA (conception between 1 January 2004 and 9 April 2012). The base cohort included 103,753 women who smoked in 140,913 pregnancies; of those, 13,667 pregnancies were excluded. Following the selection of one pregnancy per woman, data analyses included 233, 1057 and 330 women who were exposed to bupropion, varenicline and NRT, respectively, and 96,255 unexposed women (Fig. 1). As presented in Fig. 2, most women had only one dispensing of a smoking cessation therapy. Therapy initiation before conception was more common for bupropion (65.1%) and varenicline (65.6%) than for NRT (20%).

Table 1 shows that the matching procedures resulted in well-balanced baseline characteristics between exposed and unexposed pregnancies (see Additional file 2: Tables S2.1-S2.5 for before-matching characteristics). Nevertheless, in comparison to NRT-exposed women, varenicline-exposed women were more likely to be born overseas, had a partner and a private health insurance but were less likely to have a mental health condition and drug and alcohol disorders.

The analyses of the first primary outcome (Table 2) showed that the risk of having any adverse perinatal event was not significantly different between bupropion-exposed and unexposed pregnancies (hazard ratio [HR] 0.93, 95% confidence interval [CI] 0.73 to 1.19) or between NRT-exposed and unexposed pregnancies (HR 1.02, 95% CI 0.84 to 1.23). Compared to unexposed women, there was a lower risk of any adverse perinatal event in those who were exposed to varenicline (HR 0.86, 95% CI 0.77 to 0.97). When exposure to NRT was the reference group, exposure to varenicline was not associated with higher risk of any adverse event (multivariable HR 0.58, 95% CI 0.33 to 1.05, univariable HR 0.59, 95% CI 0.38 to 0.91). As presented in Table 3, 2.9% of infants exposed to varenicline in the first trimester had a major congenital anomaly compared to 3.5% of infants not exposed to any smoking cessation therapy (HR 0.91, 95% CI 0.72 to 1.15).

**Table 2:** Adverse perinatal outcomes in exposed women and the matched comparison group, stratified by therapy comparison. Data presented are numbers (percentage) of outcomes and hazard ratios (95% confidence interval)

	Aim 1									Aim 2		
	Bupropion vs unexposed			Varenicline vs unexposed			NRT vs unexposed			Varenicline vs NRT		
	Bupropion	Unexposed^#^	HR (95% CI)^†^	Varenicline	Unexposed^#^	HR (95% CI)^†^	NRT	Unexposed^#^	HR (95% CI)^†^	Varenicline	NRT^#^	HR (95% CI)
Any adverse outcome	91 (39.2%)	912 (39.3%)	0.93 (0.73–1.19)	390 (36.9%)	4240 (40.1%)	0.86 (0.77–0.97)	147 (44.8%)	1520 (46.3%)	1.02 (0.84–1.23)	67 (38.7%)	89 (51.4%)	0.58 (0.33–1.05)^‡^
Preterm	12 (5.2%)	192 (8.3%)	0.62 (0.34–1.11)	69 (6.5%)	941 (8.9%)	0.72 (0.56–0.92)	36 (11.0%)	358 (10.9%)	1.00 (0.71–1.42)	6 (3.5%)	21 (12.1%)	0.29 (0.12–0.71)^†^
Small for gestation age*	37 (16.2%)	369 (16.1%)	0.89 (0.60–1.30)	119 (11.4%)	1612 (15.4%)	0.68 (0.56–0.83)	47 (14.4%)	578 (17.7%)	0.77 (0.56–1.07)	26 (15.3%)	25 (14.7%)	1.09 (0.48–2.47)^†^
Admission to special neonatal care*	32 (14.0%)	325 (14.2%)	0.90 (0.62–1.32)	163 (15.6%)	1622 (15.5%)	0.95 (0.80–1.13)	66 (20.2%)	692 (21.2%)	0.97 (0.74–1.26)	22 (12.9%)	38 (22.4%)	0.59 (0.32–1.10)^†^
Severe neonatal morbidity complications*	15 (6.6%)	157 (6.9%)	0.87 (0.50–1.52)	69 (6.6%)	855 (8.2%)	0.74 (0.57–0.96)	27 (8.3%)	345 (10.6%)	0.82 (0.55–1.23)	13 (7.6%)	19 (11.2%)	0.60 (0.26–1.37)^†^
Emergency caesarean	34 (14.7%)	289 (12.5%)	1.15 (0.78–1.71)	123 (11.6%)	1310 (12.4%)	0.88 (0.71–1.08)	37 (11.3%)	421 (12.8%)	1.01 (0.70–1.45)	20 (11.6%)	26 (15.0%)	0.47 (0.20–1.09)^†^
Severe maternal morbidity complications	5 (2.2%)	49 (2.1%)	0.95 (0.37–2.43)	17 (1.6%)	172 (1.6%)	0.92 (0.53–1.59)	10 (3.0%)	79 (2.4%)	1.22 (0.60–2.46)	6 (3.5%)	6 (3.5%)	0.75 (0.17–3.35)^†^
PPROM	< 5	73 (3.1%)	–	36 (3.4%)	360 (3.4%)	1.02 (0.72–1.44)	16 (4.9%)	139 (4.2%)	1.15 (0.68–1.94)	< 5	9 (5.2%)	–
Apgar 5 min < 7*	< 5	44 (1.9%)	–	14 (1.3%)	288 (2.8%)	0.47 (0.27–0.81)	6 (1.8%)	105 (3.2%)	0.59 (0.25–1.37)	< 5	5 (2.9%)	–
Placenta abruption	< 5	15 (0.6%)	–	9 (0.9%)	97 (0.9%)	0.97 (0.49–1.93)	< 5	34 (1.0%)	–	0 (0%)	0 (0%)	–
Perinatal death	< 5	19 (0.8%)	–	13 (1.2%)	122 (1.2%)	1.01 (0.57–1.80)	< 5	30 (0.9%)	–	< 5	< 5	–

*Among live births

^#^Reference group

^†^For aim 1, hazard ratio was obtained from the univariate model. For aim 2, multivariable model did not converge; thus, univariate hazard ratio was reported

^‡^Hazard ratio was obtained from the multivariable Cox model which further adjusted for maternal age, having a partner, private health insurance, socio-economic disadvantage, previous caesarean section, prior hospitalisation, mental health, anaemia and coagulation disorders, and drug and alcohol disorders. In the matched sample, these co-variables had standardised difference > 0.10 and frequency ≥ 5

**Table 3:** Major congenital anomalies in infants exposed to varenicline in the first trimester and matched comparison group. Data presented are numbers (percentage) of outcomes and hazard ratios (95% confidence interval)

	Varenicline, 1st trimester	Unexposed	Hazard ratio (95% CI)^†^
Any major congenital anomaly	20 (2.9%)	242 (3.5%)	0.91 (0.72–1.15)
Genitourinary	7 (1.0%)	67 (1.0%)	1.00 (0.67–1.51)
Cardiovascular	6 (0.9%)	52 (0.7%)	1.16 (0.75–1.79)
Musculoskeletal	< 5	57 (0.8%)	–
Gastrointestinal	< 5	28 (0.4%)	–
Nevers	< 5	18 (0.3%)	–
Respiratory	< 5	6 (0.1%)	–
Eyes	< 5	11 (0.2%)	–
Face, neck	0	< 5	–
Integumentary	0	< 5	–
Genetic syndromes	< 5	7 (0.1%)	–
Situs inversus	0	< 5	–
Others	< 5	9 (0.1%)	–

^†^Hazard ratio was obtained from the univariate model

Our analyses of individual birth outcomes (Table 2) also showed that varenicline-exposed women were less likely than unexposed women to have a baby who was born preterm (HR 0.72, 95% CI 0.56 to 0.92), was small for gestational age (HR 0.68, 95% CI 0.56 to 0.83), had an Apgar score at 5 min less than 7 (HR 0.47, 95% CI 0.27 to 0.81) and had severe morbidity complications (HR 0.74, 95% CI 0.57 to 0.96). Varenicline-exposed women were also less likely than NRT-exposed women to have a preterm birth (HR 0.29, 95% CI 0.12 to 0.71).

In the sensitivity analyses restricted to women who initiated therapy after week 4 of gestation, there were large reductions in the number of exposed pregnancies. Hazard ratios for the composite adverse perinatal event were similar to those of the main analyses; however, the effects of varenicline (compared to no therapy) became statistically non-significant (see Additional file 2: Table S2.6).

## Discussion

### Principal findings

Overall, our population-based study found significant reduction in the risk of any adverse perinatal event associated with pregnancy exposure to varenicline and no increased risk of major congenital anomalies associated with first trimester exposure to varenicline. There was also no significant increase in risk of any adverse perinatal event associated with exposure to bupropion and NRT. When individual perinatal outcomes were examined, findings were most encouraging for varenicline such as a significant reduction in the risk of preterm birth, SGA and severe neonatal morbidity complications.

### Comparison with other studies

This is the first controlled comparison of varenicline exposure in pregnancy; prior studies were not able to draw a causal relationship [11–13]. Protective effects of varenicline in this study could be potentially explained by it being the most efficacious pharmacotherapy for smoking cessation as reported by studies in non-pregnant adults [40], and the timing of its use. In our study, the majority of varenicline-exposed pregnancies had the therapy dispensed prior to conception or in the first few weeks of the first trimester, a pattern that is consistent with the recommendation that varenicline not be used in pregnancy [2, 9, 10]. Early use of varenicline in pregnancy could have resulted in early quitting in pregnancy. Prior studies have shown that earlier smoking cessation in pregnancy is associated with a greater reduction in the risk of preterm birth [41, 42] and SGA [41, 43]. Whilst the early use of varenicline may explain why pregnancies exposed to varenicline had better outcomes than pregnancies not exposed to any therapy, superior efficacy of varenicline in non-pregnant adults compared to NRT [40] may explain why pregnancies exposed to varenicline had more favourable outcomes than those exposed to NRT, because in the varenicline-NRT comparisons, pregnancies were matched on gestational age at exposure. Within the Smoking MUMS Study, another analysis is underway to compare the effectiveness of varenicline and NRT when used in pregnancy.

Our study found that exposure to varenicline in the first trimester was not associated with increased risk of any major congenital anomaly. A previous study of varenicline was limited to two cases with congenital anomalies [13]. In our study, the proportion of infants with a major congenital anomaly (exposed to varenicline or exposed to smoking but not pharmacotherapy) are consistent with the underlying Australian and international figures [44–46]. To date, there were no human studies that have examined the pharmacokinetics of varenicline in pregnancy. Animal studies found that maternal exposure to high dosage of varenicline was associated with low foetal weight and development toxicity in offspring [47]. Studies in rats and rabbits did not find a teratogenic effect of varenicline, even with administered dosages 23 and 50 times higher, respectively, than the maximum recommended human daily dose [47]. However, it has been long established that animal studies are seriously limited in their ability to predict human teratogenesis [48, 49].

Overall, this is the most rigorous study to assess safety of bupropion for smoking cessation in pregnancy. Previous smaller studies [14–17] were based on self-reported exposure [16, 17] and were not able to separate bupropion use for smoking cessation from its use for depression [16, 17]. Our study found a 15% increase in the risk of emergency caesarean section although this was not statistically significant. This raised questions about the biological pathway of bupropion, given that bupropion and its metabolites can cross the placenta into foetal circulation [50].

The medicinal form of nicotine prevents a foetus from being exposed to a multitude of toxic substances in cigarette smoke [2, 9]. As nicotine and cotinine (nicotine’s metabolite) pass through the placenta [21, 51], nicotine obtained through the patches could pose health risk to the foetus, e.g. disrupted development of foetal cholinergic system in the first trimester [51]. However, due to faster clearance of nicotine and cotinine in pregnancy [52], effects of NRT would be less profound than cigarette smoke. Our study found no differences in perinatal outcomes between NRT-exposed and unexposed pregnancies which are generally consistent with reports from clinical trials [1, 18] and observational studies [19, 20]. A previous study which reported an increased risk of preterm birth and low birthweight [21] failed to control for the effect of maternal smoking. Nevertheless, a clinical trial has showed that high-dose NRT patches may increase diastolic blood pressure in late pregnancy, which may potentially lead to unfavourable pregnancy outcomes [18].

### Strengths and limitations of the study

To our knowledge, this is the largest study to date about the safety of varenicline in pregnancy. We used different study designs (i.e. non-user and active comparator) to examine outcomes associated with varenicline use; this addressed concerns about confounding by indication and health-seeking behaviours. The use of propensity score matching also addresses confounding by maternal characteristics [53]. Exposure was analysed in a time-dependant manner, eliminating immortal time bias [54]. Reliable recording of date of delivery and gestational age in perinatal data [55, 56] enabled accurate ascertainment of pregnancy exposure.

This study has some limitations. The study included pregnancies delivered at least 20 weeks of gestation; thus, outcomes such as pregnancy loss or termination before week 20 were not examined. Our study had inadequate statistical power to address effects of exposure to bupropion and NRT on rare perinatal adverse outcomes such as severe maternal morbidity complications, PPROM, Apgar score at 5 min < 7, placental abruption and perinatal mortality. It was not possible for the study to examine specific major congenital anomalies associated with first trimester varenicline exposure. Outcomes such as changes in mood, behaviour or thinking were not examined in this study. Although the risk of these mental health side effects associated with varenicline or bupropion use has been found to be less profound than previously thought, a risk remains in people with a history of mental illness [57]. In Australia, NRT can be purchased over the counter, and in geographically remote regions, clients of Aboriginal Health Services may receive NRT free of charge without the need for a prescription [58]. Such data were not captured in PBS dispensing data; thus, some exposed pregnancies would have been classified as unexposed. Nevertheless, we believe the extent of misclassification of exposure status would be negligible due to financial barriers such as low income among a large proportion of smoking women [59] and high out-of-pocket costs of NRT purchased over the counter [60]. Although the main analyses in this study assumed that exposed women took all of the dispensed medicine as per recommended dosages, this may not be the case and actual exposure status and periods of exposure may deviate from this assumption. A particular concern was that women might have ceased therapy when they became aware of their pregnancy, but sensitivity analyses focused on women who initiated therapy after week 4 of gestation suggest that the study findings are robust.

## Conclusions

Given the uncertainty about the safety of varenicline and bupropion during pregnancy, these therapies are not recommended in pregnant women. As evidence regarding the safety of NRT during pregnancy is inconsistent, clinical guidelines place the onus on the physician to decide whether the potential benefits of NRT use during pregnancy outweigh the risk of harm [2, 9, 10]. As a result, women and healthcare providers are currently in a bind when deciding whether a smoking cessation pharmacotherapy should be used, and if so, which one. Our study showed that varenicline is being used in pregnancy and its benefit is likely to outweigh the harm. This could help assess whether experimental studies might be ethical. Further multi-jurisdictional collaborations are needed for more robust evidence, which could allow investigations of outcomes such as miscarriage, terminations, specific malformations, maternal cardiovascular and neuropsychiatric events, and long-term outcomes for the babies given concerns about neurological development [47].

## Supplementary information

**Additional file 1:** Description of study variables, missing data and exclusion.

**Additional file 2:** Maternal characteristics before and after matching, results of sensitivity analyses.

## Data Availability

The data sets were constructed with the permission of each of the source data custodians and with specific ethical approvals. The authors do not have permission to share patient-level data because of the highly confidential nature of the data. Permission to access to the data is restricted to researchers named and approved by relevant Human Research Ethics Committees.

## Abbreviations

ACHI: Australian Classification of Health Interventions, Eighth Edition
ATC: Anatomical Therapeutic Chemical
HR: Hazard ratio
ICD-10-AM: International Statistical Classification of Diseases and Related Health Problems, Tenth Revision, Australian Modification
NRT: Nicotine replacement therapy
NSC: Neonatal special care
NSW: New South Wales
PBS: Pharmaceutical Benefits Scheme
PPROM: Preterm premature rupture of membranes
SGA: Small for gestational age
WA: Western Australia
