# Supplementary material for: Use of smoking cessation pharmacotherapies during pregnancy is not associated with increased risk of adverse pregnancy outcomes: a population-based cohort study
Source: BMC Med. 2020 Feb 5;18:15. doi: 10.1186/s12916-019-1472-9 (PMC7001233; doi:10.1186/s12916-019-1472-9)
Supplement: Supplementary file 1 — Additional file 1: Description of study variables, missing data and exclusion. [file 12916_2019_1472_MOESM1_ESM.docx]

## Additional File 1: Description of study variables, missing data and exclusion

The current paper used linked records from four sources including perinatal data (deliveries in 2003-2012), dispensing data for pharmaceuticals subsidised through the Pharmaceutical Benefits Scheme (PBS) (2003-2013), hospital admissions (2001-2013) and deaths (2003-2014). Data dictionaries for the NSW and WA perinatal, hospital admission and death can be obtained from the NSW Centre for Health Record Linkage [1] and WA Data Linkage Branch [2] respectively. The PBS data dictionary can be obtained from the Sax Institute [3]. Supplement 1 describes maternal characteristics, perinatal outcomes and major congenital anomalies derived from these data sources.

For PBS data source, the Anatomical Therapeutic Chemical (ATC) [4] codes and PBS item numbers were used to identify medications of interest. For hospital admission data source, diagnoses were identified from the principal and 20 additional diagnosis fields coded according to the International Statistical Classification of Diseases and Related Health Problems, Tenth Revision, Australian Modification (ICD-10-AM) [5]. Hospital procedures were identified from the principal and 10 additional procedure fields, coded according to the Australian Classification of Health Interventions, Eighth Edition (ACHI) [6]. In NSW and WA, for each singleton child birth, there was one record generated in the perinatal data (referred to as ***perinatal record***), one record generated in the hospital data for the mother (referred to as ***woman’s delivery admission***), and one record generated in the hospital data for the baby (referred to as ***baby’s birth admission***) [7]. In hospital data source, the woman’s delivery admission and baby’s birth admission were identified and linked across to individual perinatal record [7].

### Demographic and socio-economic characteristics

- **Date of conception:** Date of conception was calculated as: date of delivery – gestational age at delivery (in weeks) *7 +14 days. Both date of delivery and gestational age (i.e. the number of completed weeks of gestation) were recorded in perinatal data. Year of conception was derived from the calculated conception date.
- **Maternal age at conception**: Maternal age at conception was calculated as: maternal age at delivery (in years)– gestational age at delivery (in weeks)/52.
- **Aboriginal and/or Torres Strait Islander status**: A woman was reported as Aboriginal and Torres Strait Islander if she was recorded as Aboriginal and/or Torres Strait Islander in any of her perinatal or hospital records.
- **Country of birth**: A woman’s country of birth information was obtained from her most recent perinatal record. If country of birth was missing or inadequately described in this perinatal record, it was supplemented by information recorded in her delivery admission. Country of birth was categorised into Australia-born and overseas-born.
- **Have a partner**: For NSW, marital status was only available in the hospital data, thus it was obtained from a woman’s delivery admission. For WA, marital status information was available in both perinatal and hospital data but there was a minor degree of disparity between these two sources, thus perinatal data was used. Marital status was categorised into whether a woman had a partner (married, de-facto relationship) or did not have a partner (never married, widowed, divorced, separated).
- **Private health insurance**: Private health insurance (Yes/No) was derived from the health insurance status recorded in a woman’s delivery admission.
- **Socio-economic disadvantage of residential area**: In perinatal data, a woman’s residential area was coded and assigned to Statistical Local Area (SLA) according to the Australian Standard Geographical Classification [8]. Scores of Relative Socio-economic Disadvantage (IRSD) index based on the 2006 Australian Census of Population and Housing were mapped to the woman’s SLAs and categorised into quintiles (quintile 1 indicates the highest level of socio-economic disadvantage and quintile 5 indicates the lowest level of socio-economic disadvantage) [9].
- **Geographical remoteness of residence**: Categories of geographical remoteness (major cities, inner regional, outer regional, remote, and very remote), according to the Accessibility and Remoteness Index of Australia Plus [10] were mapped to a woman’s SLA.
- **Overseas visitor or interstate resident**: Australia has Reciprocal Health Agreement with some other countries. Under this arrangement, visitors from these countries may receive Medicare-funded medically necessary care while vising Australia [11]. Overseas visitor was identified from patient-payment information recorded in a woman’s delivery admission in hospital data. A woman was identified as an interstate resident if her geocoded SLA was in states or territories other than NSW and WA.

### Obstetric and health related characteristics

- **Parity:** Parity was recorded in perinatal data as the number of previous pregnancies greater than 20 weeks and was numerically coded (e.g. 0, 1, 2, 3). Parity was categorised into nulliparous (parity=0), multiparous (parity 1 to 4) and grand multiparous (parity >=5) [12].
- **Previous caesarean section:** This was defined as whether a woman had a caesarean section in any of her previous deliveries (Yes/No) and was recorded in the perinatal data.
- **Pregnancy interval:** Interval between two consecutive pregnancies was calculated using the equation: date of conception – date of prior delivery – 7 days [7].
- **Number of hospital admissions in 12 months prior to conception:** This was derived from a woman’s hospital data as the frequency of hospital admissions occurring within a 365-day lookback period (i.e. a period of 365 days prior to the estimated date of conception).
- **Morbidities**: Pre-existing maternal morbidities were identified from hospital and PBS data using a 365-day lookback period before conception and during the pregnancy. A previous study has shown that there was a substantial increase in the ascertainment of maternal chronic conditions from hospital records in the gestation period (i.e. conception to delivery) versus the lookback period [13]. This reflected a possibility that chronic conditions were generally managed in settings other than hospitals; thus information was not recorded in hospital data until women gave birth or get admitted due to complications in pregnancy. Therefore, to ascertain some maternal chronic morbidities (e.g. chronic bronchitis, chronic congestive heart failure), this study used admissions in both periods (365-day lookback and gestation). Pre-existing diabetes [14] and hypertension [15, 16] were also recorded in the perinatal records, thus perinatal data were used to supplement the identification of these two conditions. Classification of maternal morbidities and ICD-10-AM codes were based on previous studies [13, 17], and the grouping of medications was based on the Rx-Risk Comorbidity Index [18] (See Table S1.1).

#### Table S1.1: Ascertainment of pre-existing maternal morbidities from hospital and PBS dispensing data

| **Morbidities** | **Hospital ICD-10-AM codes (periods of the admission indicated below)** | **PBS dispensing ATC codes (365-day lookback period)** |
| --- | --- | --- |
| Mental health | Lookback and gestation periods:  F31-F34, F38, F39, F40, F41, F44, F48, F20-F25, F28-F30, O99.5 | Anxiety: N05BA01 - N05BA12, N05BE01  Bipolar: N05AN01*  Depression: N06AA01-N06AG02, N06AX03 - N06AX11, N06AX13 - N06AX18, N06AX21 - N06AX26  Psychotic illness: N05AA01 - N05AB02, N05AB06 - N05AL07, N05AX07 - N05AX13 |
| Chronic airway | Look back and gestation periods: J32, J35, J37, J40, J41, J42, J43, J44, J47, R05, O99.5  Lookback period only: J45, J46, J98, J99, | Chronic airways disease: R03AC02 - R03DC03, R03DX05 |
| Gastro-oesophageal reflux | Lookback period only: K21.0, K21.9 | Gastro-oesophageal reflux disease: A02BA01 - A02BX05 |
| Use of NSAIDS |  | NSAIDS for pain: M01AB01 - M01AH06 |
| Use of steroids |  | Steroid responsive diseases: H02AB01 - H02AB10 |
| Anaemia and coagulation | Lookback and gestation periods: D56-D57, D65-D68, D50-D53, D55, D58-D64 | Anticoagulants: B01AA03 - B01AB06, B01AE07, B01AF01, B01AF02, B01AX05  Antiplatelets: B01AC04 - B01AC07, B01AC12 - B01AC30, PBS items (05030R, 05035B, 05042J, 10111E, 10117L, 10129D, 10130E, 05751Q, 06456T) ^†^ |
| Drug and alcohol disorder | Lookback and gestation period: F10, Z50.2, Z72.1, F11, F12, F13, F14, F15, F16, F18, F19, Z50.3, Z72.2 | * Alcohol dependency: N07BB01 - N07BB99 |
| Thyroid | Lookback only: E00-E07, E89.0 | Hyperthyroidism: H03BA02 - H03BB01  Hypothyroidism: H03AA01 - H03AA02 |
| Cardiovascular | Lookback and gestation period: I05-I09, I34-I39, I50, I20, I25, I27, I28, Q20-Q25, O99.4  Lookback period only: I00-I02, I21-I24, I26, I30-I33, I40-I43, I44-I49, I51-I52, I60-I64, G45.8, G45.9, I65, I66, I67.2, I70, I73, I74, I77 | Congestive heart failure: C03DA02 - C03DA99, C07AB07, C07AB12, C07AG02, [(C03CA01 - C03CC01) and (C09AA01- C09AX99 or C09CA01 - C09CX99)], PBS items (08732N, 08733P, 08734Q, 08735R) ^‡^  Ischaemic heart disease-hypertension: C07AA01 - C07AA06, C07AA08 - C07AB01, [C07AB02 if PBS item code is not (08732N, 08733P, 08734Q, 08735R) ^‡^], C07AB03, C07AG01, C08CA01 - C08DB01, C09BB02 - C09BB10, C09DB01 - C09DB04, C09DX01, C09DX03, C10BX03 ^§^ |
| Pre-existing diabetes | Lookback and gestation period: E10, E11, E13, E14, O24.0, O24.1, O24.2, O24.3, supplemented by perinatal data ^£^ |  |
| Pre-existing hypertension | Lookback and gestation period I10, I11, I12, I13, I15,  supplemented by perinatal data ^£^ |  |
| Epilepsy | Lookback period only: G40, F80.3 | Epilepsy: N03AA01 - N03AX99 |
| Chronic renal disease | Lookback and gestation period: N02-N08, N1-N12, N14-N16, N18-N19, N25-N28, Q60-Q63, N39.1, N39.2, T82.4, T86.1, Z49, Z94.0, Z99.2  Lookback period only: N00, N01, N17 | Renal disease: A11CC01 - A11CC04, B03XA01 - B03XA03, V03AE02, V03AE03, V03AE05 |
| * Lithium (WHO N05AN01 code) was recorded as N06AX in PBS data [19]  †: These PBS items are epoprostenol and iloprost.  ‡: These PBS items are metoprolol succinate.  § Combination product for hyperlipidaemia and ischaemic heart disease: hypertension  £: Perinatal record supplements the identification of pre-existing diabetes and hypertension if these conditions were not recorded in hospital records. | | |

### Perinatal outcomes

Individual birth outcomes (categorised as Yes or No) were derived from information recorded in the perinatal record, woman’s delivery admission, baby’s birth admission, and mortality data.

- **Preterm birth**: Preterm birth was derived from gestational age at delivery and onset of labour (both recorded in perinatal data) and defined as gestational age less than 37 weeks, either medically indicated or spontaneous.
- **Small for gestational age (SGA)**: SGA was defined as birthweight <10^th^ birth weight percentile, sex- and gestational-age specific according to Australian national birthweight percentiles for singletons [20].
- **Apgar score at 5 minutes <7**: Apgar scores (recorded in perinatal data) are clinical indicators of a baby’s condition shortly after birth. A score at 5 minutes after birth <7 indicates presence of a complication for the baby [21].
- **Baby admission to neonatal special care (NSC):** Admission to NSC was recorded in the perinatal data.
- **Severe neonatal morbidity complications**: This outcome was measured by the validated Neonatal Adverse Outcome Indicator using information in the perinatal record, and baby’s birth admission and subsequent transfers to other hospitals before first discharge home [22].
- **Emergency caesarean section**: For NSW, this outcome was derived based on mode of delivery (i.e. caesarean section) and onset of labour (i.e. spontaneous or induced). For WA, this outcome was recorded in the perinatal record.
- **Severe maternal morbidity complications**: This outcome was measured by the validated Maternal Morbidity Outcome Indicator, using information recorded in a woman’s delivery admission [23].
- **Preterm premature rupture of membranes (PPROM):** This outcome was ascertained from a woman’s admissions using ICD-10-AM code O42 when gestational age at birth was <37 weeks.
- **Placental abruption**: This outcome was ascertained from a woman’s delivery admissions using ICD-10-AM code O45.
- **Perinatal death**: Perinatal death was defined as either stillbirth or neonatal death. Stillbirth was identified from baby discharge status (stillbirth, died in hospital, discharged, transferred, and other status) recorded in the perinatal data. Neonatal deaths included liveborn babies who died in hospital and those who died within 28 days from date of birth. Date of death was obtained from mortality data.

### Major congenital anomalies

In accordance with ethical approvals for this study, congenital anomalies were examined among infants born in NSW only. Among NSW live-born babies, major congenital anomalies were ascertained from their records of hospital admissions occurring within 18 months from birth, using ICD-10-AM codes and ACHI procedure codes indicative of major congenital anomalies. Initially, we compiled a list of relevant codes published by the Centre for Epidemiology and Evidence, NSW Ministry of Health (NSW Centre for Epidemiology and Evidence) [24]. We found that in our study, a small number of infants’ hospital records contained some ICD-10-AM codes listing in the ICD-10 Chapter XVII (i.e. Congenital malformations, deformations and chromosomal abnormalities Q00-Q99) [5] but these codes were not described in the NSW Centre for Epidemiology and Evidence’s aforementioned report. This might relate to disparity in data availability (i.e. the NSW Centre for Epidemiology and Evidence report was based on only one-year 2009–2010 of hospital data while our study captured up to 12 years of hospital data). For those additional and less frequently recorded ICD-10-AM codes, we subsequently followed the guidelines of the European Surveillance of Congenital Anomalies (EuroCAT) [25] to define whether they indicate major congenital anomalies (See Table S1.2). Major congenital anomalies identified from the data were classified into broad categories of body systems [24, 25]. According to the NSW Centre for Epidemiology and Evidence, the recording of major congenital anomalies in NSW hospital data for live-born children had high and very high sensitivity (at least 80%), except for the category ‘Other and unspecified anomalies’ (sensitivity 47%) [24].

#### Table S1.2: ICD-10-AM diagnosis and ACHI procedure codes used to identify major congenital anomalies and broad body system categories

| **Broad body system** | **ICD-10-AM diagnosis and ACHI procedure codes based on the NSW Centre for Epidemiology and Evidence** [24] | **ICD-10-AM diagnosis codes based on EuroCAT guidelines** [25] |
| --- | --- | --- |
| **Nervous system** | Q00.09, Q01.0, Q01.2, Q01.81, Q01.89, Q01.9, Q02, Q03.0, Q03.1, Q03.8, Q03.9, Q04.01, Q04.09, Q04.2, Q04.33, Q04.34, Q04.35, Q04.39, Q04.4, Q04.5, Q04.60, Q04.8, Q04.9, Q05.11, Q05.20, Q05.22, Q05.40, Q05.60, Q05.70, Q05.71, Q05.72, Q05.80, Q05.81, Q05.82, Q05.90, Q05.92, Q06.1, Q06.2, Q06.8, Q06.9, Q07.0, Q07.89, Q07.9 | Q01.1, Q01.82, Q01.83, Q03.01, Q03.81, Q04.00, Q04.1, Q04.31, Q04.36, Q05.00, Q05.10, Q05.12, Q05.21, Q05.30, Q05.31, Q05.32, Q05.41, Q05.42, Q05.50, Q05.52, Q05.61, Q05.62, Q06.4, Q07.81 |
| **Eye** | Q07.82, Q10.4, Q11.1, Q11.2, Q11.3, Q12.0, Q13.0, Q13.2, Q13.4, Q13.5, Q13.8, Q13.9, Q14.0, Q14.1, Q14.2, Q14.3, Q14.8, Q15.0, Q15.8, Q15.9 | Q11.0, Q12.2, Q12.3, Q12.8, Q13.1, Q13.3, Q13.41, Q13.49 |
| **Ear, face and neck** | Q16.1, Q16.4, Q16.5, Q16.9, Q17.8, Q17.9 | Q16.0, Q16.2, Q16.3 |
| **Cardiovascular system** | Q20.0, Q20.1, Q20.3, Q20.4, Q20.5, Q20.8, Q20.81, Q20.89, Q21.00, Q21.01, Q21.02, Q21.09, Q21.10, Q21.11, Q21.12, Q21.19, Q21.2, Q21.20, Q21.3, Q21.4, Q21.8, Q21.9, Q22.0, Q22.1, Q22.3, Q22.42, Q22.5, Q22.6, Q22.8, Q22.9, Q23.01, Q23.02, Q23.21, Q23.22, Q23.4, Q23.8, Q23.9, Q24.0, Q24.1, Q24.2, Q24.3, Q24.4, Q24.5, Q24.9, Q25.1, Q25.10, Q25.2, Q25.3, Q25.4, Q25.43, Q25.5, Q25.6, Q25.7, Q25.8, Q26.2, Q26.3, Q26.9, Q27.1, Q27.3, Q27.8, Q27.9, Q28.2, Q28.3, Q28.9 | Q20.2, Q20.30, Q20.31, Q20.40, Q20.41, Q20.42, Q20.49, Q20.50, Q20.59, Q20.82, Q21.0, Q21.03, Q21.1, Q21.13, Q21.21, Q21.22, Q21.24, Q21.29, Q21.83, Q22.30, Q22.31, Q22.39, Q22.4, Q22.41, Q22.81, Q22.82, Q23.0, Q23.2, Q23.81, Q23.82, Q23.83, Q24.83, Q24.86, Q24.87, Q25.11, Q25.12, Q25.13, Q25.19, Q25.30, Q25.31, Q25.39, Q25.40, Q25.44, Q25.47, Q25.49, Q25.70, Q25.79, Q26.01, Q26.02, Q26.4, Q26.5, Q26.8, Q26.81, Q28.0, Q28.1, Q28.30, Q28.31, Q28.39 |
| **Respiratory system** | Q30.0, Q30.02, Q30.2, Q30.8, Q30.9, Q31.0, Q31.1, Q31.8, Q32.1, Q32.4, Q33.01, Q33.2, Q33.8, Q33.9, Q34.8, Q34.9 Q30.01 | Q30.01, Q30.1, Q31.2, Q32.3, Q33.3, Q34.0 |
| **Gastrointestinal system** | Q35.1, Q35.11, Q35.12, Q35.3, Q35.30, Q35.31, Q35.32, Q35.5, Q35.9, Q36.0, Q36.1, Q36.9, Q37.0, Q37.1, Q37.2, Q37.3, Q37.4, Q37.5, Q37.8, Q37.9, Q38.3, Q38.4, Q38.5, Q38.6, Q38.8, Q39.0, Q39.11, Q39.12, Q39.19, Q39.21, Q39.3, Q40.2, Q40.3, Q41.0, Q41.01, Q41.1, Q41.2, Q41.9, Q42.1, Q42.20, Q42.21, Q42.22, Q42.29, Q42.3, Q42.8, Q42.9, Q43.10, Q43.11, Q43.12, Q43.19, Q43.31, Q43.32, Q43.39, Q43.5, Q43.6, Q44.1, Q44.2, Q44.3, Q44.5, Q44.71, Q44.79, Q45.1, Q45.39 | Q35.10, Q35.13, Q35.33, Q38.02, Q38.32, Q38.52, Q38.59, Q39.10, Q39.13, Q39.14, Q39.15, Q39.22, Q39.4, Q40.23, Q40.25, Q40.29, Q40.8, Q41.02, Q41.11, Q41.12, Q41.13, Q41.21, Q41.22, Q41.8, Q41.81, Q42.01, Q42.02, Q42.03, Q42.04, Q42.09, Q43.13, Q43.4, Q43.7, Q44.0, Q44.7, Q45.0, Q45.31, Q45.83 |
| **Genitourinary system** | Q50.00, Q51.3, Q52.2, Q52.6, Q52.7, Q52.8, Q52.9, Q53.0, Q54.0, Q54.1, Q54.2, Q54.3, Q54.4, Q54.8, Q54.9, Q55.00, Q55.01, Q55.02, Q55.1, Q55.22, Q55.29, Q55.4, Q55.5, Q56.4, Q60.0, Q60.2, Q60.3, Q60.4, Q60.5, Q60.6, Q61.1, Q61.3, Q61.40, Q61.41, Q61.42, Q61.8, Q62.0, Q62.11, Q62.14, Q62.18, Q62.2, Q62.31, Q62.32, Q62.34, Q62.39, Q62.51, Q62.59, Q62.62, Q62.8, Q63.01, Q63.11, Q63.9, Q64.0, Q64.19, Q64.21, Q64.31, Q64.32, Q64.75, Q64.79   Undescended testes: presence of an ICD10AM code :Q53.19, Q53.29, Q53.99, Q53.1, Q53.10, Q53.11, Q53.12, Q53.13, Q53.2, Q53.20, Q53.21, Q53.22, Q53.23, Q53.9, Q53.90, Q53.91, Q53.92, Q53.93   and an ACHI procedure: 37803-00, 37803-01 (repair of undescended testes | Q51.0, Q51.1, Q51.2, Q51.5, Q51.7, Q51.8, Q52.0, Q52.1, Q52.41, Q53.00, Q53.02, Q53.03, Q53.09, Q55.40, Q55.41, Q55.42, Q55.43, Q56.0, Q56.1, Q56.41, Q60.1, Q61.2, Q61.43, Q61.44, Q61.45, Q62.12, Q62.13, Q62.17, Q62.19, Q62.30, Q62.35, Q62.60, Q62.61, Q62.63, Q62.69, Q63.09, Q63.12, Q63.19, Q64.11, Q64.20, Q64.22, Q64.33, Q64.34, Q64.39, Q64.5, Q64.51, Q64.73, Q64.74, Q64.76 |
| **Musculoskeletal system** | Q65.0, Q65.1, Q65.2, Q65.3, Q65.4, Q65.60, Q65.61, Q65.62, Q67.49, Q67.59, Q67.6, Q67.7, Q67.8, Q68.1, Q69.0, Q69.1, Q69.21, Q69.29, Q69.9, Q70.0, Q70.2, Q70.4, Q70.9, Q71.1, Q71.31, Q71.32, Q71.33, Q71.4, Q71.5, Q71.8, Q71.9, Q72.31, Q72.4, Q72.6, Q72.7, Q72.8, Q72.9, Q73.89, Q74.07, Q74.09, Q74.3, Q75.01, Q75.02, Q75.03, Q75.04, Q75.09, Q75.1, Q75.81, Q75.89, Q75.9, Q76.39, Q76.42, Q76.43, Q76.45, Q76.61, Q76.62, Q76.69, Q76.71, Q76.79, Q77.1, Q77.2, Q77.4, Q77.7, Q78.0, Q78.8, Q78.89, Q79.0, Q79.1, Q79.2, Q79.3, Q79.4, Q79.5, Q79.6, Q79.8, Q79.9   Talipes: presence of an ICD10AM code: Q66.0, Q66.00, Q66.01, Q66.02, Q66.1, Q66.4 and an ACHI procedure: 49718-01, 49724-00, 49724-01, 49727-00, 50321-00, 50324-00, 50324-01, 50327-00 (for repair of talipes) | Q71.12, Q71.2, Q71.41, Q72.0, Q72.2, Q72.32, Q72.33, Q74.04, Q74.4, Q74.85, Q75.05, Q75.06, Q75.4, Q76.1, Q76.21, Q76.31, Q76.33, Q76.34, Q76.41, Q76.46, Q77.02, Q77.03, Q77.3, Q77.6, Q77.82, Q78.1, Q78.2, Q78.5, Q78.82, Q79.11, Q79.12, Q79.84 |
| **Integumentary system** | D18.1, Q80.9, Q81.8, Q81.9, Q82.1, Q82.3, Q83.1, Q83.8, Q83.9, Q84.0, Q84.3, Q84.81, Q85.1, Q85.81, Q85.82 | Q80.0, Q80.1, Q80.2, Q80.3, Q80.4, Q80.8, Q81.0, Q81.1, Q81.2, Q82.0, Q82.81, Q82.82, Q83.2, Q85.84 |
| **Congenital malformation syndromes** | D82.1, Q87.04, Q87.06, Q87.09, Q87.12, Q87.13, Q87.14, Q87.17, Q87.19, Q87.21, Q87.22, Q87.27, Q87.31, Q87.32, Q87.4, Q87.5, Q87.82, Q87.84, Q87.86, Q87.87, Q87.89 | Q87.00, Q87.02, Q87.03, Q87.07, Q87.08, Q87.11, Q87.15, Q87.16, Q87.18, Q87.23, Q87.24, Q87.25, Q87.26, Q87.81, Q87.83, Q87.85, Q87.88 |
| **Situs inversus** | Q89.30, Q89.31, Q89.39 | Q89.32, Q89.33, Q89.34, Q89.35 |
| **Other and unspecified anomalies** | E03.0, E03.1, E70.0, E70.1, E84.0, E84.1, E84.8, E84.9, P83.2, Q20.6, Q89.01, Q89.09, Q89.12, Q89.19, Q89.21, Q89.26, Q89.29, Q89.79, Q89.89, Q89.9 | D21.5, Q89.00, Q89.02, Q89.05, Q89.10, Q89.11, Q89.14, Q89.22, Q89.25, Q89.81, Q89.82 |
| **Chromosomal anomalies** | Q90.9, Q91.0, Q91.3, Q91.5, Q91.7, Q92.8, Q92.9, Q93.3, Q93.4, Q93.5, Q93.8, Q96.3, Q96.4, Q96.8, Q96.9, Q97.0, Q97.3, Q98.0, Q98.1, Q98.4, Q99.1, Q99.8, Q99.9 | Q90.0, Q90.1, Q90.2, Q91.1, Q91.2, Q91.4, Q91.6, Q92.1, Q92.3, Q92.5, Q92.6, Q92.7, Q92.71, Q93.1, Q93.2, Q93.7, Q93.9, Q96.0, Q96.1, Q97.1, Q97.2, Q97.8, Q98.5, Q98.6, Q98.7, Q98.8, Q98.9, Q99, Q99.0, Q99.2 |
| **Congenital viral infection due to rubella, cytomegalovirus, herpes** | P35.0, P35.1, P35.2 |  |
| **Congenital due to specified exogenous causes** | Q86.0, Q86.1, Q86.81, Q86.82, Q86.85, Q86.89 |  |

### Missing data

#### Table S1.3: Number of pregnancies with missing information

| **Information** | **Number**  **(out of 140,913 pregnancies in the base cohort)*** |  |
| --- | --- | --- |
| Statistical Local Area of residence ^†^ | 476 |  |
| Maternal age (conception) | 4 |  |
| Aboriginal and Torres Strait Islander identity | 11 |  |
| Mode of delivery ^‡^ | 49 |  |
| Parity | 37 |  |
| Gestational age ^††^ | 3 |  |
| Birth weight ^††^ | 57 |  |
| Baby’s sex ^††^ | 31 |  |
| Apgar score at 5 minutes | 483 |  |
| Type of maternity facility | 78 |  |
| **Total number of pregnancies with any missing data** | **1217** |  |
| *: Base cohort included pregnancies conceived between 1 January 2004 and 9 April 2012 during which a woman smoked  ^†^: Statistical Local Area of residence recorded in perinatal data was used to derive socio-economic disadvantage and geographic remoteness of the woman’s residential area.  ^‡^: Mode of delivery and onset of labour recorded in perinatal data were used to derive the outcome variable emergency caesarean section  ^††^: Gestational age, birth weight and baby’s sex were used to derive the outcome variable small for gestational age. | | |

### Exclusion

#### Table S1.4: Number of pregnancies excluded, according to exclusion criteria

| **Exclusion criteria** | **Number**  **(out of 140,913 pregnancies in the base cohort)*** |  |
| --- | --- | --- |
| Multiple births | 1905 |  |
| Pregnancy interval<6 months | 6526 |  |
| Overseas visitors or interstate residents (likely incomplete capture of hospital admission and dispensing data) | 1599 |  |
| Use of multiple smoking therapies in the same pregnancy | 5 |  |
| Use of potentially teratogenic medications during pregnancy (category D and X, according to the Australian Therapeutic Goods Administration classification system) [26] | 2400 |  |
| Likely data error (i.e. birthweight <1000g whilst gestational age >38 weeks, birthweight >5500g whilst gestational age <37 week, based on the Australian birthweight chart) [20] | 15 |  |
| Missing data | 1217 |  |
| Pregnancies linked to congenital anomalies due to chromosomal malformations, viral infections and known exogenous causes were further excluded ^†^ | 119 |  |
| *: Base cohort included pregnancies conceived between 1 January 2004 and 9 April 2012 during which a woman smoked  ^†^: These 119 pregnancies were only excluded from the analyses examining the outcome major congenital anomaly. | | |

## References for Additional File 1

1. Centre for Health Record Linkage. Data dictionaries 2018. <http://www.cherel.org.au/data-dictionaries>.

2. Western Australia Data Linkage Branch. Specific information for core datasets: Western Australia Data Linkage Branch; 2016. <http://www.datalinkage-wa.org.au/downloads/dataset-information>.

3. The Sax Institute. PBS data dictionary Sydney, Australia2017. <https://www.saxinstitute.org.au/wp-content/uploads/PBS-Data-Dictionary-January-2017.pdf>. Accessed 4 April 2019.

4. World Health Organization. Anatomical Therapeutic Chemical (ATC) Classification 2019. <https://www.who.int/medicines/regulation/medicines-safety/toolkit_atc/en/>. Accessed 4 April 2019.

5. National Centre for Classification in Health. The International Statistical Classification of Diseases and Related Health Problems, Tenth Revision, Australian Modification (ICD-10-AM)-Fifth Edition-Tabular list of diseases and Alphabetic index of diseases. Sydney: National Centre for Classification in Health, Faculty of Health Sciences, The University of Sydney; 2008.

6. National Centre for Classification in Health. The Australian Classification of Health Interventions (ACHI) – Seventh Edition - Tabular list of interventions and Alphabetic index of interventions. Sydney: National Centre for Classification in Health, Faculty of Health Sciences, The University of Sydney; 2010.

7. Tran DT, Havard A, Jorm LR. Data cleaning and management protocols for linked perinatal research data: a good practice example from the Smoking MUMS (Maternal Use of Medications and Safety) Study. BMC Med Res Methodol. 2017;17(1):97.

8. Australian Bureau of Statistics. 1216.0 - Australian Standard Geographical Classification (ASGC), July 2011: Commonwealth of Australia; 2010. <https://www.abs.gov.au/AUSSTATS/abs@.nsf/Latestproducts/DEDA554E1B6BB78BCA25791F000EEA26>. Accessed 15 April 2019.

9. Australian Bureau of Statistics. 2033.0.55.001. Census of Population and Housing: Socio-Economic Indexes for Areas (SEIFA), Australia 2006: Australian Bureau of Statistics; 2008. <http://www.abs.gov.au/AUSSTATS/abs@.nsf/Lookup/2033.0.55.001Main+Features12006?OpenDocument>. Accessed 5 February 2018.

10. Australian Bureau of Statistics. Australian Standard Geographic Classification Remoteness Structure: Commonwealth of Australia; 2010. <http://www.abs.gov.au/websitedbs/D3310114.nsf/home/remoteness+structure>. Accessed 15 April 2019.

11. Australian Government Department of Human Services. Medical care for visitors to Australia 2019. <https://www.humanservices.gov.au/individuals/services/medicare/reciprocal-health-care-agreements/visitors-australia/medical-care-visitors-australia#a8>. Accessed 4 April 2019.

12. Australian Institute of Health and Welfare. Maternal deaths in Australia 2012–2014. Canberra: Australian Institute of Health and Welfare; 2017.

13. Chen JS, Roberts C, Simpson J, Ford J. Use of hospitalisation history (lookback) to determine prevalence of chronic diseases: Impact on modelling of risk factors for haemorrhage in pregnancy. BMC Med Res Methodol. 2011;11(1):68.

14. Bell JC, Ford JB, Cameron CA, Roberts CL. The accuracy of population health data for monitoring trends and outcomes among women with diabetes in pregnancy. Diabetes Res Clin Pract. 2008;81(1):105-9.

15. Roberts CL, Algert CS, Morris JM, Ford JB, Henderson-Smart DJ. Hypertensive disorders in pregnancy: a population-based study. MJA. 2005;182(7):332-5.

16. Roberts CL, Bell JC, Ford JB, Hadfield RM, Algert CS, Morris JM. The accuracy of reporting of the hypertensive disorders of pregnancy in population health data. Hypertens Pregnancy. 2008;27(3):285-97.

17. Metcalfe A, Lix LM, Johnson JA, Currie G, Lyon AW, Bernier F, et al. Validation of an obstetric comorbidity index in an external population. BJOG. 2015;122(13):1748-55.

18. Pratt NL, Kerr M, Barratt JD, Kemp-Casey A, Kalisch Ellett LM, Ramsay E, et al. The validity of the Rx-Risk Comorbidity Index using medicines mapped to the Anatomical Therapeutic Chemical (ATC) Classification System. BMJ Open. 2018;8(4):e021122.

19. Australian Institute of Health and Welfare. Mental health services in Australia: Data sources. Canberra: Australian Institute of Health and Welfare; 2019.

20. Dobbins TA, Sullivan EA, Roberts CL, Simpson JM. Australian national birthweight percentiles by sex and gestational age, 1998-2007. MJA. 2012;197(5):291-4.

21. Australian Institute of Health and Welfare. Australia’s mothers and babies — in brief. Canberra: AIHW; 2018.

22. Lain SJ, Algert CS, Nassar N, Bowen JR, Roberts CL. Incidence of severe adverse neonatal outcomes: Use of a composite indicator in a population cohort. Matern Child Health J. 2012;16(3):600-8.

23. Roberts CL, Cameron CA, Bell JC, Algert CS, Morris JM. Measuring maternal morbidity in routinely collected health data: development and validation of a maternal morbidity outcome indicator. Med Care. 2008;46(8):786-94.

24. Centre for Epidemiology and Evidence. Quality and coverage of the NSW Register of Congenital Conditions using Admitted Patient Data: A record linkage study Sydney: NSW Ministry of Health; 2016. <https://www.health.nsw.gov.au/hsnsw/Pages/rocc-apd-linkage-study.aspx>. Accessed 4 April 2018.

25. European Surveillance of Congenital Anomalies. EUROCAT Guide 1.4 and Reference Documents (Version 28/12/2018). Section 3.3. EUROCAT Subgroups of Congenital Anomalies 2018. <https://eu-rd-platform.jrc.ec.europa.eu/sites/default/files/Full_Guide_1_4_version_28_DEC2018.pdf>. Accessed 4 April 2019.

26. Australian Therapeutic Goods Administration. Reporting adverse events: Australian Department of Health; 2018. <https://www.tga.gov.au/reporting-adverse-events#faq>. Accessed 25 October 2019.
