# Supplementary material for: Use of smoking cessation pharmacotherapies during pregnancy is not associated with increased risk of adverse pregnancy outcomes: a population-based cohort study
Source: BMC Med. 2020 Feb 5;18:15. doi: 10.1186/s12916-019-1472-9 (PMC7001233; doi:10.1186/s12916-019-1472-9)
Supplement: Supplementary file 2 — Additional file 2: Maternal characteristics before and after matching, results of sensitivity analyses. [file 12916_2019_1472_MOESM2_ESM.docx]

## Additional File 2: Maternal characteristics before and after matching, results of sensitivity analyses

### Table S2.1: Maternal characteristics of smoking women who were exposed to bupropion and who were not exposed to any pharmacotherapies during pregnancy: Numbers (percentage) and standardised differences, before and after propensity score matching

|  | | **Before matching** | | |  | **After matching** | | |
| --- | --- | --- | --- | --- | --- | --- | --- | --- |
|  |  | Bupropion | Unexposed | Diff * |  | Bupropion | Unexposed | Diff * |
| **Total number** | | **233** | **96255** |  |  | **232** | **2320** |  |
| **State** | |  |  |  |  |  |  |  |
|  | New South Wales | 178 (76.4%) | 69185 (71.9%) | 0.10 |  | 177 (76.3%) | 1819 (78.4%) | 0.05 |
|  | Western Australia | 55 (23.6%) | 27070 (28.1%) | 0.10 |  | 55 (23.7%) | 501 (21.6%) | 0.05 |
| **Year of conception** | |  |  |  |  |  |  |  |
|  | 2004 | 39 (16.7%) | 16474 (17.1%) | 0.01 |  | 39 (16.8%) | 389 (16.8%) | 0.00 |
|  | 2005 | 59 (25.3%) | 15814 (16.4%) | 0.22 |  | 59 (25.4%) | 613 (26.4%) | 0.02 |
|  | 2006 | 42 (18.0%) | 13751 (14.3%) | 0.10 |  | 42 (18.1%) | 391 (16.9%) | 0.03 |
|  | 2007 | 46 (19.7%) | 11885 (12.3%) | 0.20 |  | 45 (19.4%) | 483 (20.8%) | 0.04 |
|  | 2008 | 17 (7.3%) | 10951 (11.4%) | 0.14 |  | 17 (7.3%) | 194 (8.4%) | 0.04 |
|  | 2009 | 7 (3.0%) | 9539 (9.9%) | 0.28 |  | 7 (3.0%) | 75 (3.2%) | 0.01 |
|  | 2010 | 12 (5.2%) | 8311 (8.6%) | 0.14 |  | 12 (5.2%) | 93 (4.0%) | 0.06 |
|  | 2011+2012 | 11 (4.7%) # | 9530 (9.9%) # | 0.20 |  | 11 (4.7%) # | 82 (3.6%) # | 0.05 |
| **Maternal age (conception)** | |  |  |  |  |  |  |  |
|  | Under 25 | 61 (26.2%) | 42268 (43.9%) | 0.38 |  | 61 (26.3%) | 589 (25.4%) | 0.02 |
|  | 25-29 | 76 (32.6%) | 23959 (24.9%) | 0.17 |  | 76 (32.8%) | 792 (34.1%) | 0.03 |
|  | 30-34 | 62 (26.6%) | 18944 (19.7%) | 0.16 |  | 61 (26.3%) | 602 (25.9%) | 0.01 |
|  | 35 and older | 34 (14.6%) | 11084 (11.5%) | 0.09 |  | 34 (14.7%) | 337 (14.5%) | 0.00 |
| **Aboriginal** | | 9 (3.9%) | 15231 (15.8%) | 0.41 |  | 9 (3.9%) | 110 (4.7%) | 0.04 |
| **Overseas born** | | 36 (15.5%) | 13736 (14.3%) | 0.03 |  | 35 (15.1%) | 367 (15.8%) | 0.02 |
| **Having a partner** | | 173 (74.2%) | 58382 (60.7%) | 0.29 |  | 173 (74.6%) | 1764 (76.0%) | 0.03 |
| **Private health insurance** | | 34 (14.6%) | 8206 (8.5%) | 0.19 |  | 34 (14.7%) | 288 (12.4%) | 0.07 |
| **Socio-economic disadvantage ‡** | |  |  |  |  |  |  |  |
|  | Quintile 1 (most) | 49 (21.0%) | 18945 (19.7%) | 0.03 |  | 48 (20.7%) | 519 (22.4%) | 0.04 |
|  | Quintile 2 | 39 (16.7%) | 17416 (18.1%) | 0.04 |  | 39 (16.8%) | 411 (17.7%) | 0.02 |
|  | Quintile 3 | 46 (19.7%) | 21815 (22.7%) | 0.07 |  | 46 (19.8%) | 445 (19.2%) | 0.02 |
|  | Quintile 4 | 54 (23.2%) | 23495 (24.4%) | 0.03 |  | 54 (23.3%) | 524 (22.6%) | 0.02 |
|  | Quintile 5 (least) | 45 (19.3%) | 14584 (15.2%) | 0.11 |  | 45 (19.4%) | 421 (18.1%) | 0.03 |
| **Remoteness of residence** | |  |  |  |  |  |  |  |
|  | Major cities | 136 (58.4%) | 51278 (53.3%) | 0.10 |  | 135 (58.2%) | 1374 (59.2%) | 0.02 |
|  | Inner regional | 63 (27.0%) | 29704 (30.9%) | 0.08 |  | 63 (27.2%) | 598 (25.8%) | 0.03 |
|  | Outer regional, remote | 34 (14.6%) | 15273 (15.9%) | 0.04 |  | 34 (14.7%) | 348 (15.0%) | 0.01 |
| **Parity** | |  |  |  |  |  |  |  |
|  | Nulliparous | 65 (27.9%) | 40202 (41.8%) | 0.29 |  | 65 (28.0%) | 699 (30.1%) | 0.05 |
|  | Multiparous (1 to 4) | 153 (65.7%) | 51564 (53.6%) | 0.25 |  | 152 (65.5%) | 1466 (63.2%) | 0.05 |
|  | Grand multiparous (>=5) | 15 (6.4%) | 4489 (4.7%) | 0.08 |  | 15 (6.5%) | 155 (6.7%) | 0.01 |
| **Previous caesarean section** | | 33 (14.2%) | 11436 (11.9%) | 0.07 |  | 33 (14.2%) | 290 (12.5%) | 0.05 |
| **Hospitalisation, 12 months prior** | |  |  |  |  |  |  |  |
|  | Nil | 187 (80.3%) | 77593 (80.6%) | 0.01 |  | 187 (80.6%) | 1901 (81.9%) | 0.03 |
|  | Once | 35 (15.0%) | 13819 (14.4%) | 0.02 |  | 35 (15.1%) | 327 (14.1%) | 0.03 |
|  | Two or more | 11 (4.7%) | 4843 (5.0%) | 0.01 |  | 10 (4.3%) | 92 (4.0%) | 0.02 |
| **Morbidities** | |  |  |  |  |  |  |  |
|  | Mental health | 58 (24.9%) | 16461 (17.1%) | 0.19 |  | 57 (24.6%) | 534 (23.0%) | 0.04 |
|  | Chronic airway | 46 (19.7%) | 10445 (10.9%) | 0.25 |  | 45 (19.4%) | 416 (17.9%) | 0.04 |
|  | Gastro-oesophageal reflux | 17 (7.3%) | 2729 (2.8%) | 0.20 |  | 16 (6.9%) | 149 (6.4%) | 0.02 |
|  | Use of NSAIDS | 24 (10.3%) | 3518 (3.7%) | 0.26 |  | 23 (9.9%) | 206 (8.9%) | 0.04 |
|  | Use of steroids | 12 (5.2%) | 1342 (1.4%) | 0.21 |  | 11 (4.7%) | 92 (4.0%) | 0.04 |
|  | Anaemia and coagulation | 11 (4.7%) | 3800 (3.9%) | 0.04 |  | 10 (4.3%) | 108 (4.7%) | 0.02 |
|  | Drug and alcohol | 7 (3.0%) | 7359 (7.6%) | 0.21 |  | 7 (3.0%) | 59 (2.5%) | 0.03 |
|  | Thyroid | <5 | 547 (0.6%) | 0.08 |  | <5 | 21 (0.9%) | 0.04 |
|  | Cardiovascular | <5 | 906 (0.9%) | 0.06 |  | <5 | 5 (0.2%) | 0.04 |
|  | Pre-existing diabetes | <5 | 618 (0.6%) | 0.03 |  | <5 | 12 (0.5%) | 0.01 |
|  | Pre-existing hypertension | <5 | 717 (0.7%) | 0.01 |  | <5 | 12 (0.5%) | 0.04 |
|  | Epilepsy | <5 | 936 (1.0%) | 0.07 |  | <5 | 9 (0.4%) | 0.01 |
|  | Chronic renal disease | <5 | 761 (0.8%) | 0.01 |  | <5 | 27 (1.2%) | 0.03 |
| *: Absolute standardised difference. #: Data for 2011 and 2012 were combined due to frequency of women exposed to bupropion in 2012 was <5. ‡: Quintile scores of the Index of Relative Socio-economic Disadvantage, based on residential area of women.  <5: In accordance with ethical approvals, frequencies less than 5 were not reported. | | | | | | | | |
|  |  |  |  |  |  |  |  |  |

### Table S2.2: Maternal characteristics of smoking women who were exposed to varenicline and who were not exposed to any pharmacotherapies during pregnancy: Numbers (percentage) and standardised differences, before and after propensity score matching

|  | | **Before matching** | | |  | **After matching** | | |
| --- | --- | --- | --- | --- | --- | --- | --- | --- |
|  |  | Varenicline | Unexposed | Diff * |  | Varenicline | Unexposed | Diff * |
| **Total number** | | **1057** | **52729** |  |  | **1057** | **10570** |  |
| **State** | |  |  |  |  |  |  |  |
|  | New South Wales | 801 (75.8%) | 38013 (72.1%) | 0.08 |  | 801 (75.8%) | 8109 (76.7%) | 0.02 |
|  | Western Australia | 256 (24.2%) | 14716 (27.9%) | 0.08 |  | 256 (24.2%) | 2461 (23.3%) | 0.02 |
| **Year of conception** | |  |  |  |  |  |  |  |
|  | 2008 | 179 (16.9%) | 15567 (29.5%) | 0.30 |  | 179 (16.9%) | 1699 (16.1%) | 0.02 |
|  | 2009 | 270 (25.5%) | 13716 (26.0%) | 0.01 |  | 270 (25.5%) | 2666 (25.2%) | 0.01 |
|  | 2010 | 315 (29.8%) | 11314 (21.5%) | 0.19 |  | 315 (29.8%) | 3153 (29.8%) | 0.00 |
|  | 2011 | 239 (22.6%) | 9843 (18.7%) | 0.10 |  | 239 (22.6%) | 2480 (23.5%) | 0.02 |
|  | 2012 | 54 (5.1%) | 2289 (4.3%) | 0.04 |  | 54 (5.1%) | 572 (5.4%) | 0.01 |
| **Maternal age (conception)** | |  |  |  |  |  |  |  |
|  | Under 25 | 332 (31.4%) | 22529 (42.7%) | 0.24 |  | 332 (31.4%) | 3341 (31.6%) | 0.00 |
|  | 25-29 | 318 (30.1%) | 13836 (26.2%) | 0.09 |  | 318 (30.1%) | 3132 (29.6%) | 0.01 |
|  | 30-34 | 227 (21.5%) | 10012 (19.0%) | 0.06 |  | 227 (21.5%) | 2360 (22.3%) | 0.02 |
|  | 35 and older | 180 (17.0%) | 6352 (12.0%) | 0.14 |  | 180 (17.0%) | 1737 (16.4%) | 0.02 |
| **Aboriginal** | | 101 (9.6%) | 9445 (17.9%) | 0.24 |  | 101 (9.6%) | 926 (8.8%) | 0.03 |
| **Overseas born** | | 180 (17.0%) | 7546 (14.3%) | 0.07 |  | 180 (17.0%) | 1815 (17.2%) | 0.00 |
| **Having a partner** | | 719 (68.0%) | 31210 (59.2%) | 0.18 |  | 719 (68.0%) | 7218 (68.3%) | 0.01 |
| **Private health insurance** | | 141 (13.3%) | 4037 (7.7%) | 0.19 |  | 141 (13.3%) | 1435 (13.6%) | 0.01 |
| **Socio-economic disadvantage ‡** | |  |  |  |  |  |  |  |
|  | Quintile 1 (most) | 172 (16.3%) | 10919 (20.7%) | 0.11 |  | 172 (16.3%) | 1722 (16.3%) | 0.00 |
|  | Quintile 2 | 164 (15.5%) | 9706 (18.4%) | 0.08 |  | 164 (15.5%) | 1629 (15.4%) | 0.00 |
|  | Quintile 3 | 263 (24.9%) | 11946 (22.7%) | 0.05 |  | 263 (24.9%) | 2546 (24.1%) | 0.02 |
|  | Quintile 4 | 289 (27.3%) | 12724 (24.1%) | 0.07 |  | 289 (27.3%) | 3024 (28.6%) | 0.03 |
|  | Quintile 5 (least) | 169 (16.0%) | 7434 (14.1%) | 0.05 |  | 169 (16.0%) | 1649 (15.6%) | 0.01 |
| **Remoteness of residence** | |  |  |  |  |  |  |  |
|  | Major cities | 592 (56.0%) | 27513 (52.2%) | 0.08 |  | 592 (56.0%) | 5927 (56.1%) | 0.00 |
|  | Inner regional | 339 (32.1%) | 16752 (31.8%) | 0.01 |  | 339 (32.1%) | 3474 (32.9%) | 0.02 |
|  | Outer regional, remote | 126 (11.9%) | 8464 (16.1%) | 0.12 |  | 126 (11.9%) | 1169 (11.1%) | 0.03 |
| **Parity** | |  |  |  |  |  |  |  |
|  | Nulliparous | 299 (28.3%) | 19856 (37.7%) | 0.20 |  | 299 (28.3%) | 3022 (28.6%) | 0.01 |
|  | Multiparous (1 to 4) | 709 (67.1%) | 29655 (56.2%) | 0.22 |  | 709 (67.1%) | 7122 (67.4%) | 0.01 |
|  | Grand multiparous (>=5) | 49 (4.6%) | 3218 (6.1%) | 0.07 |  | 49 (4.6%) | 426 (4.0%) | 0.03 |
| **Previous caesarean section** | | 175 (16.6%) | 7442 (14.1%) | 0.07 |  | 175 (16.6%) | 1663 (15.7%) | 0.02 |
| **Hospitalisation, 12 months prior** | |  |  |  |  |  |  |  |
|  | Nil | 856 (81.0%) | 42576 (80.7%) | 0.01 |  | 856 (81.0%) | 8655 (81.9%) | 0.02 |
|  | Once | 151 (14.3%) | 7432 (14.1%) | 0.01 |  | 151 (14.3%) | 1418 (13.4%) | 0.03 |
|  | Two or more | 50 (4.7%) | 2721 (5.2%) | 0.02 |  | 50 (4.7%) | 497 (4.7%) | 0.00 |
| **Morbidities** | |  |  |  |  |  |  |  |
|  | Mental health | 229 (21.7%) | 9155 (17.4%) | 0.11 |  | 229 (21.7%) | 2162 (20.5%) | 0.03 |
|  | Chronic airway | 168 (15.9%) | 5687 (10.8%) | 0.15 |  | 168 (15.9%) | 1419 (13.4%) | 0.07 |
|  | Gastro-oesophageal reflux | 66 (6.2%) | 1594 (3.0%) | 0.15 |  | 66 (6.2%) | 585 (5.5%) | 0.03 |
|  | Use of NSAIDS | 61 (5.8%) | 1718 (3.3%) | 0.12 |  | 61 (5.8%) | 510 (4.8%) | 0.04 |
|  | Use of steroids | 30 (2.8%) | 851 (1.6%) | 0.08 |  | 30 (2.8%) | 243 (2.3%) | 0.03 |
|  | Anaemia and coagulation | 26 (2.5%) | 2005 (3.8%) | 0.08 |  | 26 (2.5%) | 223 (2.1%) | 0.02 |
|  | Drug and alcohol | 25 (2.4%) | 3533 (6.7%) | 0.21 |  | 25 (2.4%) | 230 (2.2%) | 0.01 |
|  | Thyroid | 8 (0.8%) | 261 (0.5%) | 0.03 |  | 8 (0.8%) | 79 (0.7%) | 0.00 |
|  | Cardiovascular | 12 (1.1%) | 484 (0.9%) | 0.02 |  | 12 (1.1%) | 113 (1.1%) | 0.01 |
|  | Pre-existing diabetes | 13 (1.2%) | 367 (0.7%) | 0.05 |  | 13 (1.2%) | 114 (1.1%) | 0.01 |
|  | Pre-existing hypertension | 11 (1.0%) | 416 (0.8%) | 0.03 |  | 11 (1.0%) | 96 (0.9%) | 0.01 |
|  | Epilepsy | 8 (0.8%) | 515 (1.0%) | 0.02 |  | 8 (0.8%) | 64 (0.6%) | 0.02 |
|  | Chronic renal disease | 5 (0.5%) | 420 (0.8%) | 0.04 |  | 5 (0.5%) | 42 (0.4%) | 0.01 |
| *: Absolute standardised difference. ‡: Quintile scores of the Index of Relative Socio-economic Disadvantage, based on residential area of women.  <5: In accordance with ethical approvals, frequencies less than 5 were not reported. | | | | | | | | |
|  |  |  |  |  |  |  |  |  |

### Table S2.3: Maternal characteristics of smoking women who were exposed to NRT and who were not exposed to any pharmacotherapies during pregnancy: Numbers (percentage) and standardised differences, before and after propensity score matching

|  | | **Before matching** | | |  | **After matching** | | |
| --- | --- | --- | --- | --- | --- | --- | --- | --- |
|  |  | NRT | Unexposed † | Diff * |  | NRT | Unexposed † | Diff * |
| **Total number** | | **330** | **20462** |  |  | **328** | **3280** |  |
| **State** | |  |  |  |  |  |  |  |
|  | New South Wales | 250 (75.8%) | 14287 (69.8%) | 0.13 |  | 248 (75.6%) | 2484 (75.7%) | 0.00 |
|  | Western Australia | 80 (24.2%) | 6175 (30.2%) | 0.13 |  | 80 (24.4%) | 796 (24.3%) | 0.00 |
| **Year of conception** | |  |  |  |  |  |  |  |
|  | 2009 | 9 (2.7%) § | 2573 (12.6%) † | 0.38 |  | 9 (2.7%) | 73 (2.2%) † | 0.03 |
|  | 2010 | 48 (14.5%) | 2459 (12.0%) † | 0.07 |  | 47 (14.3%) | 412 (12.6%) † | 0.05 |
|  | 2011 | 213 (64.5%) | 12411 (60.7%) | 0.08 |  | 212 (64.6%) | 2228 (67.9%) | 0.07 |
|  | 2012 | 60 (18.2%) | 3019 (14.8%) | 0.09 |  | 60 (18.3%) | 567 (17.3%) | 0.03 |
| **Maternal age (conception)** | |  |  |  |  |  |  |  |
|  | Under 25 | 94 (28.5%) | 9648 (47.2%) | 0.39 |  | 94 (28.7%) | 910 (27.7%) | 0.02 |
|  | 25-29 | 100 (30.3%) | 5245 (25.6%) | 0.10 |  | 99 (30.2%) | 1060 (32.3%) | 0.05 |
|  | 30-34 | 84 (25.5%) | 3510 (17.2%) | 0.20 |  | 84 (25.6%) | 819 (25.0%) | 0.01 |
|  | 35 and older | 52 (15.8%) | 2059 (10.1%) | 0.17 |  | 51 (15.5%) | 491 (15.0%) | 0.02 |
| **Aboriginal** | | 95 (28.8%) | 7570 (37.0%) | 0.18 |  | 93 (28.4%) | 875 (26.7%) | 0.04 |
| **Overseas born** | | 24 (7.3%) | 2169 (10.6%) | 0.12 |  | 24 (7.3%) | 206 (6.3%) | 0.04 |
| **Having a partner** | | 179 (54.2%) | 10867 (53.1%) | 0.02 |  | 179 (54.6%) | 1792 (54.6%) | 0.00 |
| **Private health insurance** | | 23 (7.0%) | 1123 (5.5%) | 0.06 |  | 23 (7.0%) | 207 (6.3%) | 0.03 |
| **Socio-economic disadvantage ‡** | |  |  |  |  |  |  |  |
|  | Quintile 1 (most) | 54 (16.4%) | 4832 (23.6%) | 0.18 |  | 53 (16.2%) | 479 (14.6%) | 0.04 |
|  | Quintile 2 | 62 (18.8%) | 3720 (18.2%) | 0.02 |  | 62 (18.9%) | 644 (19.6%) | 0.02 |
|  | Quintile 3 | 84 (25.5%) | 4876 (23.8%) | 0.04 |  | 84 (25.6%) | 875 (26.7%) | 0.02 |
|  | Quintile 4 | 92 (27.9%) | 4538 (22.2%) | 0.13 |  | 91 (27.7%) | 873 (26.6%) | 0.03 |
|  | Quintile 5 (least) | 38 (11.5%) | 2496 (12.2%) | 0.02 |  | 38 (11.6%) | 409 (12.5%) | 0.03 |
| **Remoteness of residence** | |  |  |  |  |  |  |  |
|  | Major cities | 172 (52.1%) | 9390 (45.9%) | 0.12 |  | 170 (51.8%) | 1677 (51.1%) | 0.01 |
|  | Inner regional | 118 (35.8%) | 6680 (32.6%) | 0.07 |  | 118 (36.0%) | 1257 (38.3%) | 0.05 |
|  | Outer regional, remote | 40 (12.1%) | 4392 (21.5%) | 0.25 |  | 40 (12.2%) | 346 (10.5%) | 0.05 |
| **Parity** | |  |  |  |  |  |  |  |
|  | Nulliparous | 73 (22.1%) | 6650 (32.5%) | 0.23 |  | 73 (22.3%) | 751 (22.9%) | 0.02 |
|  | Multiparous (1 to 4) | 239 (72.4%) | 12182 (59.5%) | 0.27 |  | 237 (72.3%) | 2367 (72.2%) | 0.00 |
|  | Grand multiparous (>=5) | 18 (5.5%) | 1630 (8.0%) | 0.10 |  | 18 (5.5%) | 162 (4.9%) | 0.02 |
| **Previous caesarean section** | | 69 (20.9%) | 3105 (15.2%) | 0.15 |  | 69 (21.0%) | 626 (19.1%) | 0.05 |
| **Hospitalisation, 12 months prior** | |  |  |  |  |  |  |  |
|  | Nil | 256 (77.6%) | 16325 (79.8%) | 0.05 |  | 254 (77.4%) | 2608 (79.5%) | 0.05 |
|  | Once | 54 (16.4%) | 3022 (14.8%) | 0.04 |  | 54 (16.5%) | 502 (15.3%) | 0.03 |
|  | Two or more | 20 (6.1%) | 1115 (5.4%) | 0.03 |  | 20 (6.1%) | 170 (5.2%) | 0.04 |
| **Morbidities** | |  |  |  |  |  |  |  |
|  | Mental health | 133 (40.3%) | 3862 (18.9%) | 0.48 |  | 131 (39.9%) | 1224 (37.3%) | 0.05 |
|  | Chronic airway | 57 (17.3%) | 2388 (11.7%) | 0.16 |  | 57 (17.4%) | 516 (15.7%) | 0.04 |
|  | Gastro-oesophageal reflux | 14 (4.2%) | 573 (2.8%) | 0.08 |  | 13 (4.0%) | 137 (4.2%) | 0.01 |
|  | Use of NSAIDS | 24 (7.3%) | 671 (3.3%) | 0.18 |  | 23 (7.0%) | 197 (6.0%) | 0.04 |
|  | Use of steroids | 14 (4.2%) | 381 (1.9%) | 0.14 |  | 14 (4.3%) | 104 (3.2%) | 0.06 |
|  | Anaemia and coagulation | 13 (3.9%) | 1022 (5.0%) | 0.05 |  | 13 (4.0%) | 124 (3.8%) | 0.01 |
|  | Drug and alcohol | 30 (9.1%) | 1667 (8.1%) | 0.03 |  | 30 (9.1%) | 251 (7.7%) | 0.05 |
|  | Thyroid | 5 (1.5%) | 120 (0.6%) | 0.09 |  | 5 (1.5%) | 52 (1.6%) | 0.00 |
|  | Cardiovascular | 7 (2.1%) | 239 (1.2%) | 0.07 |  | 6 (1.8%) | 70 (2.1%) | 0.02 |
|  | Pre-existing diabetes | 5 (1.5%) | 178 (0.9%) | 0.06 |  | 5 (1.5%) | 45 (1.4%) | 0.01 |
|  | Pre-existing hypertension | 6 (1.8%) | 167 (0.8%) | 0.09 |  | 6 (1.8%) | 45 (1.4%) | 0.04 |
|  | Epilepsy | 10 (3.0%) | 170 (0.8%) | 0.16 |  | 8 (2.4%) | 65 (2.0%) | 0.03 |
|  | Chronic renal disease | <5 | 199 (1.0%) | 0.02 |  | <5 | 43 (1.3%) | 0.01 |
| *: Absolute standardised difference.  †: For 2009 and 2010, only Aboriginal women were included. ‡: Quintile scores of the Index of Relative Socio-economic Disadvantage, based on residential area of women.  <5: In accordance with ethical approvals, frequencies less than 5 were not reported. | | | | | | | | |
|  |  |  |  |  |  |  |  |  |

### Table S2.4: Maternal characteristics of smoking women who were exposed to varenicline in the first trimester and who were not exposed to any pharmacotherapies during pregnancy: Numbers (percentage) and standardised differences, before and after propensity score matching

|  | | **Before matching** | | |  | **After matching** | | |
| --- | --- | --- | --- | --- | --- | --- | --- | --- |
|  |  | Varenicline | Unexposed | Diff * |  | Varenicline | Unexposed | Diff * |
| **Total number** | | **696** | **37756** |  |  | **696** | **6960** |  |
| **State** | |  |  |  |  |  |  |  |
|  | New South Wales | 696 (100%) | 37756 (100%) |  |  | 696 (100%) | 6960 (100%) |  |
| **Year of conception** | |  |  |  |  |  |  |  |
|  | 2008 | 116 (16.7%) | 11090 (29.4%) | 0.31 |  | 116 (16.7%) | 1057 (15.2%) | 0.04 |
|  | 2009 | 169 (24.3%) | 9892 (26.2%) | 0.04 |  | 169 (24.3%) | 1740 (25.0%) | 0.02 |
|  | 2010 | 220 (31.6%) | 8198 (21.7%) | 0.23 |  | 220 (31.6%) | 2200 (31.6%) | 0.00 |
|  | 2011 | 155 (22.3%) | 7008 (18.6%) | 0.09 |  | 155 (22.3%) | 1643 (23.6%) | 0.03 |
|  | 2012 | 36 (5.2%) | 1568 (4.2%) | 0.05 |  | 36 (5.2%) | 320 (4.6%) | 0.03 |
| **Maternal age (conception)** | |  |  |  |  |  |  |  |
|  | Under 25 | 215 (30.9%) | 15750 (41.7%) | 0.23 |  | 215 (30.9%) | 2172 (31.2%) | 0.01 |
|  | 25-29 | 230 (33.0%) | 10013 (26.5%) | 0.14 |  | 230 (33.0%) | 2177 (31.3%) | 0.04 |
|  | 30-34 | 133 (19.1%) | 7234 (19.2%) | 0.00 |  | 133 (19.1%) | 1418 (20.4%) | 0.03 |
|  | 35 and older | 118 (17.0%) | 4759 (12.6%) | 0.12 |  | 118 (17.0%) | 1193 (17.1%) | 0.00 |
| **Aboriginal** | | 71 (10.2%) | 6061 (16.1%) | 0.17 |  | 71 (10.2%) | 648 (9.3%) | 0.03 |
| **Overseas born** | | 104 (14.9%) | 4973 (13.2%) | 0.05 |  | 104 (14.9%) | 1015 (14.6%) | 0.01 |
| **Having a partner** | | 452 (64.9%) | 21177 (56.1%) | 0.18 |  | 452 (64.9%) | 4584 (65.9%) | 0.02 |
| **Private health insurance** | | 87 (12.5%) | 2945 (7.8%) | 0.16 |  | 87 (12.5%) | 836 (12.0%) | 0.01 |
| **Socio-economic disadvantage ‡** | |  |  |  |  |  |  |  |
|  | Quintile 1 (most) | 123 (17.7%) | 8802 (23.3%) | 0.14 |  | 123 (17.7%) | 1264 (18.2%) | 0.01 |
|  | Quintile 2 | 112 (16.1%) | 7200 (19.1%) | 0.08 |  | 112 (16.1%) | 1073 (15.4%) | 0.02 |
|  | Quintile 3 | 201 (28.9%) | 9414 (24.9%) | 0.09 |  | 201 (28.9%) | 1948 (28.0%) | 0.02 |
|  | Quintile 4 | 173 (24.9%) | 8116 (21.5%) | 0.08 |  | 173 (24.9%) | 1763 (25.3%) | 0.01 |
|  | Quintile 5 (least) | 87 (12.5%) | 4224 (11.2%) | 0.04 |  | 87 (12.5%) | 912 (13.1%) | 0.02 |
| **Remoteness of residence** | |  |  |  |  |  |  |  |
|  | Major cities | 440 (63.2%) | 23308 (61.7%) | 0.03 |  | 440 (63.2%) | 4451 (64.0%) | 0.02 |
|  | Inner regional | 200 (28.7%) | 10319 (27.3%) | 0.03 |  | 200 (28.7%) | 1999 (28.7%) | 0.00 |
|  | Outer regional, remote | 56 (8.0%) | 4129 (10.9%) | 0.10 |  | 56 (8.0%) | 510 (7.3%) | 0.03 |
| **Parity** | |  |  |  |  |  |  |  |
|  | Nulliparous | 221 (31.8%) | 15908 (42.1%) | 0.22 |  | 221 (31.8%) | 2197 (31.6%) | 0.00 |
|  | Multiparous (1 to 4) | 455 (65.4%) | 20308 (53.8%) | 0.24 |  | 455 (65.4%) | 4587 (65.9%) | 0.01 |
|  | Grand multiparous (>=5) | 20 (2.9%) | 1540 (4.1%) | 0.07 |  | 20 (2.9%) | 176 (2.5%) | 0.02 |
| **Previous caesarean section** | | 109 (15.7%) | 5222 (13.8%) | 0.05 |  | 109 (15.7%) | 1048 (15.1%) | 0.02 |
| **Hospitalisation, 12 months prior** | |  |  |  |  |  |  |  |
|  | Nil | 560 (80.5%) | 30796 (81.6%) | 0.03 |  | 560 (80.5%) | 5754 (82.7%) | 0.06 |
|  | Once | 101 (14.5%) | 5087 (13.5%) | 0.03 |  | 101 (14.5%) | 870 (12.5%) | 0.06 |
|  | Two or more | 35 (5.0%) | 1873 (5.0%) | 0.00 |  | 35 (5.0%) | 336 (4.8%) | 0.01 |
| **Morbidities** | |  |  |  |  |  |  |  |
|  | Mental health | 156 (22.4%) | 6585 (17.4%) | 0.12 |  | 156 (22.4%) | 1502 (21.6%) | 0.02 |
|  | Chronic airway | 119 (17.1%) | 4382 (11.6%) | 0.16 |  | 119 (17.1%) | 1069 (15.4%) | 0.05 |
|  | Gastro-oesophageal reflux | 56 (8.0%) | 1542 (4.1%) | 0.17 |  | 56 (8.0%) | 486 (7.0%) | 0.04 |
|  | Use of NSAIDS | 54 (7.8%) | 1689 (4.5%) | 0.14 |  | 54 (7.8%) | 481 (6.9%) | 0.03 |
|  | Use of steroids | 25 (3.6%) | 841 (2.2%) | 0.08 |  | 25 (3.6%) | 239 (3.4%) | 0.01 |
|  | Anaemia and coagulation | 16 (2.3%) | 1001 (2.7%) | 0.02 |  | 16 (2.3%) | 141 (2.0%) | 0.02 |
|  | Drug and alcohol | 19 (2.7%) | 2323 (6.2%) | 0.17 |  | 19 (2.7%) | 169 (2.4%) | 0.02 |
|  | Thyroid | 8 (1.1%) | 240 (0.6%) | 0.05 |  | 8 (1.1%) | 62 (0.9%) | 0.03 |
|  | Cardiovascular | 10 (1.4%) | 335 (0.9%) | 0.05 |  | 10 (1.4%) | 87 (1.3%) | 0.02 |
|  | Pre-existing diabetes | <5 | 205 (0.5%) | 0.00 |  | <5 | 55 (0.8%) | 0.03 |
|  | Pre-existing hypertension | 6 (0.9%) | 269 (0.7%) | 0.02 |  | 6 (0.9%) | 57 (0.8%) | 0.00 |
|  | Epilepsy | 5 (0.7%) | 389 (1.0%) | 0.03 |  | 5 (0.7%) | 60 (0.9%) | 0.02 |
|  | Chronic renal disease | <5 | 259 (0.7%) | 0.01 |  | <5 | 30 (0.4%) | 0.02 |
| *: Absolute standardised difference. ‡: Quintile scores of the Index of Relative Socio-economic Disadvantage, based on residential area of women.  <5: In accordance with ethical approvals, frequencies less than 5 were not reported. | | | | | | | | |
|  |  |  |  |  |  |  |  |  |
|  |  |  |  |  |  |  |  |  |

### Table S2.5: Maternal characteristics of smoking women who were exposed to varenicline and who were exposed to NRT during pregnancy: Numbers (percentage) and standardised differences, before and after propensity score matching

|  | | **Before matching** | | |  | **After matching** | | |
| --- | --- | --- | --- | --- | --- | --- | --- | --- |
|  |  | Varenicline † | NRT | Diff * |  | Varenicline † | NRT | Diff * |
| **Total number** | | 879 | 326 |  |  | 173 | 173 |  |
| **State** | |  |  |  |  |  |  |  |
|  | New South Wales | 662 (75.3%) | 246 (75.5%) | 0.00 |  | 131 (75.7%) | 127 (73.4%) | 0.05 |
|  | Western Australia | 217 (24.7%) | 80 (24.5%) | 0.00 |  | 42 (24.3%) | 46 (26.6%) | 0.05 |
| **Year of conception** | |  |  |  |  |  |  |  |
|  | 2009+2010 | 585 (66.6%) †§ | 57 (17.5%) § | 1.15 |  | 21 (12.1%) †§ | 22 (12.7%) § | 0.02 |
|  | 2011 | 240 (27.3%) | 211 (64.7%) | 0.81 |  | 123 (71.1%) | 116 (67.1%) | 0.09 |
|  | 2012 | 54 (6.1%) | 58 (17.8%) | 0.36 |  | 29 (16.8%) | 35 (20.2%) | 0.09 |
| **Maternal age (conception)** | |  |  |  |  |  |  |  |
|  | Under 25 | 280 (31.9%) | 93 (28.5%) | 0.07 |  | 57 (32.9%) | 47 (27.2%) | 0.13 |
|  | 25-29 | 267 (30.4%) | 99 (30.4%) | 0.00 |  | 53 (30.6%) | 54 (31.2%) | 0.01 |
|  | 30-34 | 192 (21.8%) | 82 (25.2%) | 0.08 |  | 34 (19.7%) | 41 (23.7%) | 0.10 |
|  | 35 and older | 140 (15.9%) | 52 (16.0%) | 0.00 |  | 29 (16.8%) | 31 (17.9%) | 0.03 |
| **Aboriginal** | | 81 (9.2%) | 67 (20.6%) | 0.32 |  | 32 (18.5%) | 36 (20.8%) | 0.06 |
| **Overseas born** | | 147 (16.7%) | 24 (7.4%) | 0.29 |  | 26 (15.0%) | 11 (6.4%) | 0.28 |
| **Having a partner** | | 594 (67.6%) | 176 (54.0%) | 0.28 |  | 119 (68.8%) | 91 (52.6%) | 0.34 |
| **Private health insurance** | | 113 (12.9%) | 22 (6.7%) | 0.21 |  | 20 (11.6%) | 10 (5.8%) | 0.21 |
| **Socio-economic disadvantage ‡** | |  |  |  |  |  |  |  |
|  | Quintile 1 (most) | 141 (16.0%) | 54 (16.6%) | 0.01 |  | 33 (19.1%) | 26 (15.0%) | 0.11 |
|  | Quintile 2 | 135 (15.4%) | 62 (19.0%) | 0.10 |  | 23 (13.3%) | 41 (23.7%) | 0.27 |
|  | Quintile 3 | 220 (25.0%) | 81 (24.8%) | 0.00 |  | 40 (23.1%) | 37 (21.4%) | 0.04 |
|  | Quintile 4 | 239 (27.2%) | 92 (28.2%) | 0.02 |  | 44 (25.4%) | 46 (26.6%) | 0.03 |
|  | Quintile 5 (least) | 144 (16.4%) | 37 (11.3%) | 0.15 |  | 33 (19.1%) | 23 (13.3%) | 0.16 |
| **Remoteness of residence** | |  |  |  |  |  |  |  |
|  | Major cities | 492 (56.0%) | 169 (51.8%) | 0.08 |  | 96 (55.5%) | 93 (53.8%) | 0.03 |
|  | Inner regional | 285 (32.4%) | 117 (35.9%) | 0.07 |  | 57 (32.9%) | 62 (35.8%) | 0.06 |
|  | Outer regional, remote | 102 (11.6%) | 40 (12.3%) | 0.02 |  | 20 (11.6%) | 18 (10.4%) | 0.04 |
| **Parity** | |  |  |  |  |  |  |  |
|  | Nulliparous | 249 (28.3%) | 73 (22.4%) | 0.14 |  | 46 (26.6%) | 41 (23.7%) | 0.07 |
|  | Multiparous (1 to 4) | 592 (67.3%) | 235 (72.1%) | 0.10 |  | 116 (67.1%) | 122 (70.5%) | 0.07 |
|  | Grand multiparous (>=5) | 38 (4.3%) | 18 (5.5%) | 0.06 |  | 11 (6.4%) | 10 (5.8%) | 0.02 |
| **Previous caesarean section** | | 142 (16.2%) | 69 (21.2%) | 0.13 |  | 28 (16.2%) | 37 (21.4%) | 0.13 |
| **Hospitalisation, 12 months prior** | |  |  |  |  |  |  |  |
|  | Nil | 710 (80.8%) | 254 (77.9%) | 0.07 |  | 142 (82.1%) | 132 (76.3%) | 0.14 |
|  | Once | 128 (14.6%) | 52 (16.0%) | 0.04 |  | 23 (13.3%) | 27 (15.6%) | 0.07 |
|  | Two or more | 41 (4.7%) | 20 (6.1%) | 0.07 |  | 8 (4.6%) | 14 (8.1%) | 0.14 |
| **Morbidities** | |  |  |  |  |  |  |  |
|  | Mental health | 178 (20.3%) | 132 (40.5%) | 0.45 |  | 39 (22.5%) | 75 (43.4%) | 0.45 |
|  | Chronic airway | 136 (15.5%) | 56 (17.2%) | 0.05 |  | 22 (12.7%) | 28 (16.2%) | 0.10 |
|  | Gastro-oesophageal reflux | 58 (6.6%) | 14 (4.3%) | 0.10 |  | 11 (6.4%) | 8 (4.6%) | 0.08 |
|  | Use of NSAIDS | 49 (5.6%) | 24 (7.4%) | 0.07 |  | 7 (4.0%) | 9 (5.2%) | 0.06 |
|  | Use of steroids | 25 (2.8%) | 13 (4.0%) | 0.06 |  | <5 | 5 (2.9%) | 0.04 |
|  | Anaemia and coagulation | 21 (2.4%) | 13 (4.0%) | 0.09 |  | 5 (2.9%) | 9 (5.2%) | 0.12 |
|  | Drug and alcohol | 20 (2.3%) | 30 (9.2%) | 0.30 |  | 5 (2.9%) | 21 (12.1%) | 0.36 |
|  | Thyroid | 8 (0.9%) | 5 (1.5%) | 0.06 |  | <5 | <5 | 0.00 |
|  | Cardiovascular | 9 (1.0%) | 7 (2.1%) | 0.09 |  | <5 | 5 (2.9%) | 0.18 |
|  | Pre-existing diabetes | 10 (1.1%) | 5 (1.5%) | 0.03 |  | <5 | 5 (2.9%) | 0.04 |
|  | Pre-existing hypertension | 9 (1.0%) | 6 (1.8%) | 0.07 |  | <5 | <5 | 0.15 |
|  | Epilepsy | 8 (0.9%) | 10 (3.1%) | 0.15 |  | <5 | 5 (2.9%) | 0.18 |
|  | Chronic renal disease | <5 | <5 | 0.08 |  | <5 | <5 | 0.06 |
| *: Absolute standardised difference.  †: For 2009 and 2010, only Aboriginal women were included.  §: Data for 2009 and 2010 were combined due to frequency of women exposed to NRT in 2009 was <5 ‡: Quintile scores of the Index of Relative Socio-economic Disadvantage, based on residential area of women.  <5: In accordance with ethical approvals, frequencies less than 5 were not reported. | | | | | | | | |
|  |  |  |  |  |  |  |  |  |

### Table S2.6: Hazard Ratios and 95%CI for any perinatal adverse event: main analyses and sensitivity analyses

Sensitivity analyses restricted the analyses to women who initiated the therapy after week four of gestation (i.e. gestational age at exposure >4 weeks).


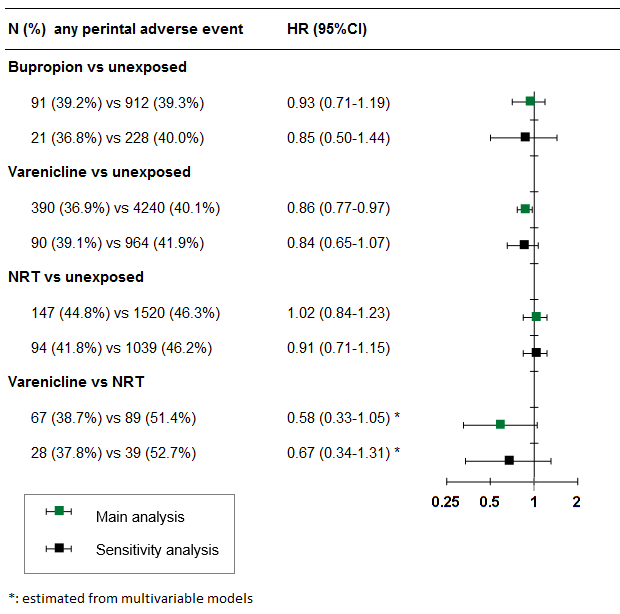


*****: Hazard ratio was obtained from multivariable Cox model which further adjusted for maternal age, having a partner, private health insurance, socio-economic disadvantage, previous caesarean section, prior hospitalisation, mental health, anaemia and coagulation disorders, and drug and alcohol disorders. In the matched samples, these co-variables had standardised difference >0.10 and frequency ≥5.
